# A Critical Quantity for Noise Attenuation in Feedback Systems

**DOI:** 10.1371/journal.pcbi.1000764

**Published:** 2010-04-29

**Authors:** Liming Wang, Jack Xin, Qing Nie

**Affiliations:** Center for Mathematical and Computational Biology, Center for Complex Biological Systems, and Department of Mathematics, University of California at Irvine, Irvine, California, United States of America; University of Illinois at Urbana-Champaign, United States of America

## Abstract

Feedback modules, which appear ubiquitously in biological regulations, are often subject to disturbances from the input, leading to fluctuations in the output. Thus, the question becomes how a feedback system can produce a faithful response with a noisy input. We employed multiple time scale analysis, Fluctuation Dissipation Theorem, linear stability, and numerical simulations to investigate a module with one positive feedback loop driven by an external stimulus, and we obtained a critical quantity in noise attenuation, termed as “signed activation time”. We then studied the signed activation time for a system of two positive feedback loops, a system of one positive feedback loop and one negative feedback loop, and six other existing biological models consisting of multiple components along with positive and negative feedback loops. An inverse relationship is found between the noise amplification rate and the signed activation time, defined as the difference between the deactivation and activation time scales of the noise-free system, normalized by the frequency of noises presented in the input. Thus, the combination of fast activation and slow deactivation provides the best noise attenuation, and it can be attained in a single positive feedback loop system. An additional positive feedback loop often leads to a marked decrease in activation time, decrease or slight increase of deactivation time and allows larger kinetic rate variations for slow deactivation and fast activation. On the other hand, a negative feedback loop may increase the activation and deactivation times. The negative relationship between the noise amplification rate and the signed activation time also holds for the six other biological models with multiple components and feedback loops. This principle may be applicable to other feedback systems.

## Author Summary

Many biological systems use feedback loops to regulate dynamic interactions among different genes and proteins. Here, we ask how interlinked feedback loops control the *timing* of signal transductions and responses and, consequently, attenuate noise. Drawing on simple modeling along with both analytical insights and computational assessments, we have identified a key quantity, termed as the “signed activation time”, that dictates a system's ability of attenuating noise. This quantity combining the speed of deactivation and activation in signal responses, relative to the input noise frequency, is determined by the property of feedback systems when noises are absent. In general, such quantity could be measured experimentally through the output response time of a signaling system driven by pulse stimulus. This principle for noise attenuation in feedback loops may also be applicable to other biological systems involving more complex regulations.

## Introduction

It has been identified that feedback loops play important roles in a variety of biological processes, such as calcium signaling [1], [2], p53 regulation [3], galactose regulation [4], cell cycle [5]–[8], and budding yeast polarization [9]–[13]. Although the detailed regulation of feedback loops may vary in different systems, the overall functions of feedback loop modules may be similar. For example, positive feedback loops are mainly used for promoting bi-stable switches and amplifying signals. One example is the cell cycle system [5]–[8] in which the mitotic regulator CDK1 activates Cdc25, which in turn activates CDK1, forming a positive feedback loop. Conversely, Wee1 and CDK1 inactivate each other, forming a double-negative feedback loop, equivalent to a positive feedback loop. The overall positive feedback regulation gives rise to a bi-stable switch that toggles between the inter-phase state and the mitotic-phase state. Another example is the system of yeast mating [9]–[15], in which multi-stage positive feedback loops enable the localization of signaling molecules at the plasma membrane by amplifying signals to initiate cell polarization and mating.

While most studies of feedback loops have been concerned with their roles in signal amplification, switch (or switch-like) responses [16]–[20], and oscillations [21] (See [22], [23] for the latest review.), recently, another important aspect of feedback loops has drawn more and more attention: modulating (accelerating or delaying) timing of signal responses [22], [24], [25]. Intuitively, positive feedback could amplify signals inducing an expeditious activation, or delay an activation by setting a higher threshold such that the system is activated only when the response accumulates beyond that threshold [22], [25]. Because characteristics of noises (e.g., the temporal frequency of a noise) in a biological process are closely related to timing of a signaling system, feedbacks clearly play a critical role in noise attenuation [26]–[29].

Thus, one of the central questions on noise analysis is how the architecture of a feedback circuit affects its noise property. Some studies suggested that positive feedbacks tended to amplify noise and negative feedbacks typically attenuated noise [30]–[32]; on the other hand, some other studies demonstrated that the positive feedbacks could attenuate noises and there were no strong correlations between the sign of feedbacks (negative or positive) and the noise attenuation properties [28], [33].

**Figure 1:**
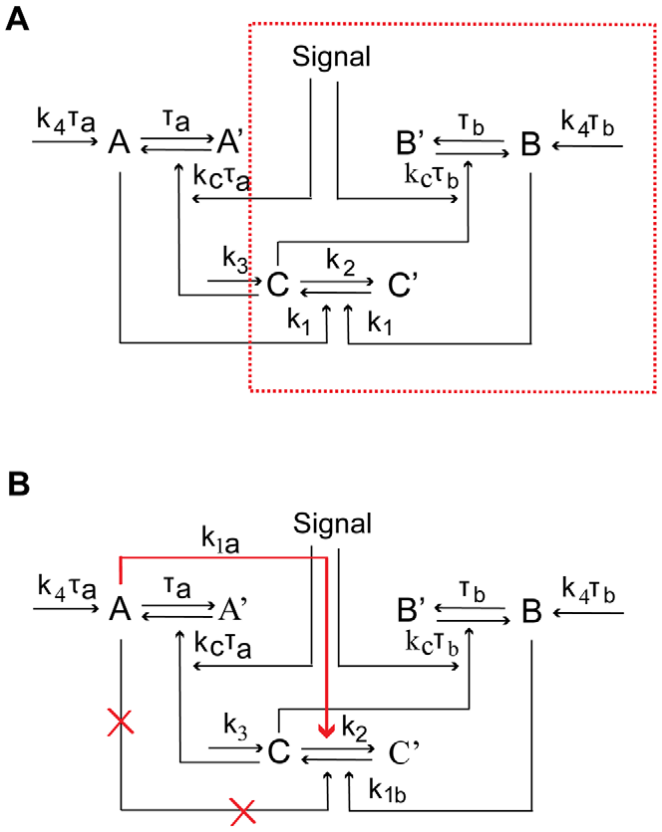
Schematic diagrams of single-positive-loop, positive-positive-loop, and positive-negative-loop modules. (A) The positive feedback modules. In this plot, there are three components: loop 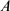, loop 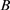, and output 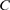. 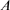, 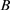, and 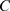 denote the active forms, whereas 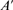, 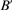, and 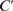 stand for the corresponding inactive forms, respectively. The red dashed box represents the single-positive-loop module consisting of 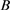 and 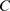 only. In the 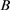 component, signals come in to active 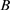 with the help of 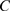 at the rate of 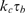. All other activation processes of 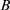 are lumped into one term, the basal activation rate 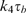. The conversion from 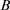 to 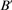 has the rate 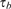. In the 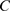 component, 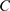 is activated by 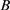 at the rate of 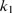, and the deactivation of 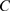 is at the rate of 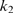. The basal activation rate of 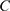 is 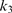. Similar notations are used in the 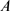 component. (B) The positive-negative-loop module. The positive feedback from 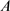 to 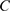 is replaced by negative feedback (red arrow).

In their novel work [34], Brandman *et al.* linked the effect of positive feedback loops on noise attenuation to the time scales of the feedback loops. They studied a canonical feedback module consisting of three components, i.e., an output 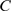 and two positive feedback loops, 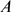 and 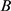. The output 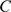 is turned on by the two positive feedback loops and 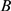, which are stimulated by an external (or upstream) stimulus and are also facilitated by 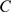 (Figure 1A). The output 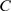 becomes active (or stays inactive) as the pulse stimulus is high (or low). Through numerical simulations, Brandman *et al.* [34] showed that, if one of the positive feedback loops (e.g., loop 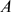) was slow and the other one was fast (termed as dual-time loops), the system could lead to distinct active output 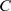 even in the presence of noise in the stimulus (at the high state).

Following this work, Zhang *et al.* [35] studied dual-time loops in producing a bi-stable response with a constant input (unlike a pulse input in [34]). They concluded that dual-time loops were the most robust design among all combinations in producing bi-stable output for a slightly different system in which the stimulus could activate 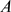 or 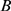 without the participation of 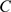. Kim *et al.* [36] considered systems coupled with negative and positive feedback loops. By assuming all the positive feedback loops have the same time scale but different time delays, they obtained a system that was capable of performing fast activation, fast deactivation, and noise attenuation.

What remains unclear are the sufficient and necessary conditions for a feedback system to achieve noise attenuation. Are two, or at least two, positive feedback loops (as used in [34]–[36]) required for controlling noise amplification in the input? Is a fast loop necessary for a positive feedback loop system to achieve noise attenuation? Are there any intrinsic quantities that connect the dynamic property of a system in absence of noises with the system's capability of noise suppression? If such quantities exist, how do positive feedbacks or negative feedbacks affect them?

In this work, we find that the capability of noise suppression in a system strongly depends on a quantity that measures the difference between the deactivation and activation times relative to the input noise frequency. Specifically, this quantity, termed as the “signed activation time”, has an inverse relationship with the noise amplification rate, with larger signed activation time leading to better noise attenuation. In addition, the signed activation time , representing one of the essential temporal characteristics of the system in absence of noises, may be controlled by either negative or positive feedbacks. We explore the properties of the quantity through both analytic approach (including linear stability analysis, multiple time scale analysis, and Fluctuation Dissipation Theorem) and numerical simulations. We first consider the same modules as in [34], and find that, for example, an additional positive feedback loop could drastically increase the signed activation time by speeding up the activation time while still keeping the deactivation time slow, as consistent with the previous observation [34] that dual-time-loop systems suppress noises better than single-loop systems. We next add a negative feedback loop to the positive-feedback-only system and show that a negative feedback loop usually slows down both activation and deactivation processes, leading to better or worse noise attenuation depending on which process (between activation and deactivation) is more significantly affected. Finally, we study the signed activation time and its relations to the noise amplification rate in different systems involving various feedbacks (e.g., positive, negative, and feedforward), including a yeast cell polarization model [14], [37], a polymyxin B resistance model in enteric bacteria [38], and four connector-mediated models [39]. All simulations confirm that the capability of noise attenuation in those systems improves as the signed activation time increases.

## Results

### The Difference between Deactivation and Activation Time Scales Dictates Noise Attenuation Ability

A simple model with one positive feedback loop may have two components with one upstream stimulus (inside the red dashed box in Figure 1A). In this system, the output 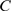 is activated by 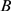, and 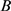 is triggered by a stimulus 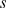 and regulated by 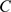. The stimulus 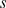 drives the output of the system with a high (or low) stimulus that corresponds to an active (or inactive) state of 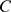. Many biological circuits have positive feedback regulations of this nature [1], [2], [19], [40]. For example, 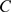 is a kinase to phosphorylate 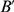 to 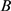, and once 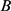 is activated, it catalyzes a conversion from an inactive form 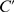 to an active form 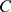 [5]. Neglecting the mechanistic details, while keeping the essential interactions, we model the dynamics of the above module by the following system of ordinary differential equations (Text S1):
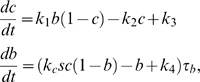
(1)where 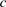 and 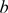 represent normalized concentrations of 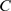 and 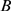, respectively. The normalized stimulus 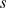, as a function of time, 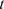, usually varies (continuously) between two states, i.e., an inactive state in which 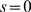 (or the “off” state) and an active state in which 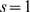 (or the “on” state). The parameters 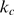, 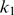, 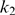, and 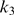 are kinetic constants, and 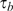 indicates the time scale for loop 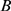.

**Figure 2:**
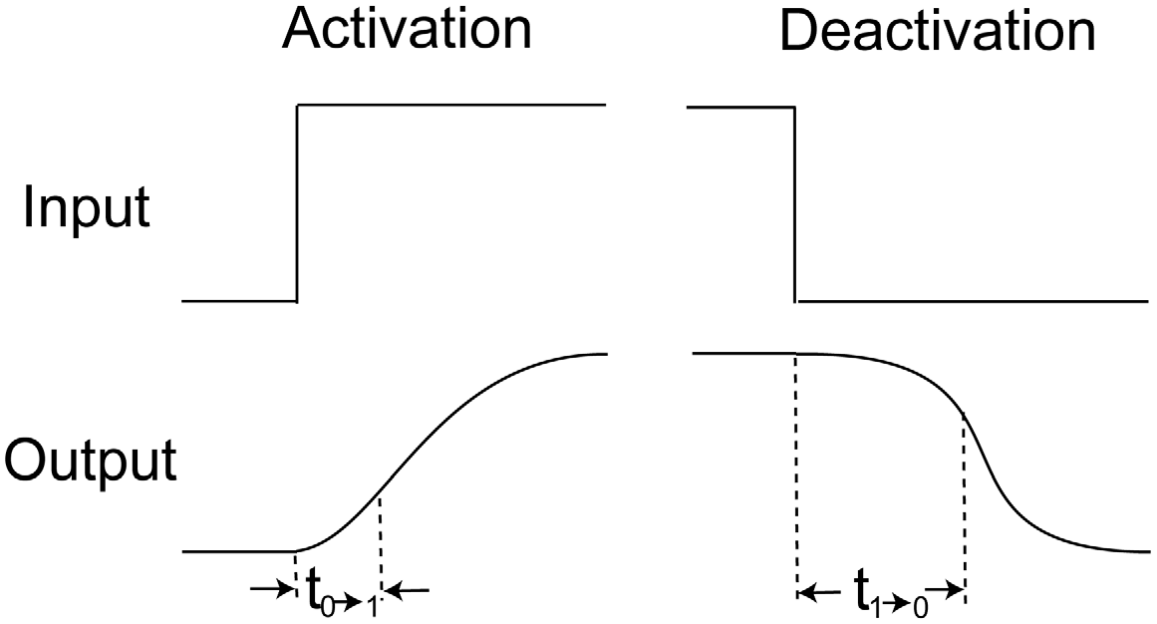
Schematic illustration of the activation (left) and deactivation (right) time scales.

**Figure 3:**
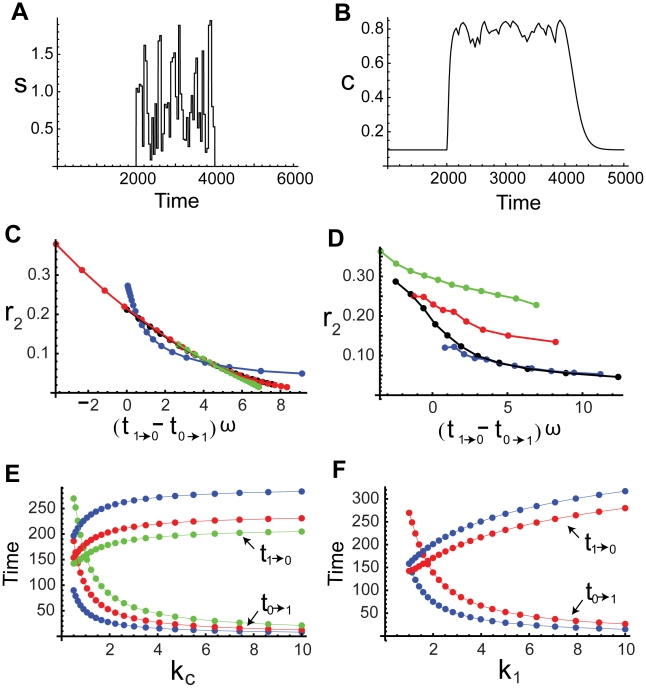
Noise attenuation and time scales in single-positive-loop systems. (A) A noisy signal with frequency 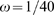 and 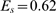 (defined in Methods). (B) A typical output response to the signal in (A). (C) 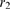 versus 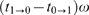. Kinetic parameters 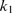 (black), 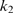 (red), 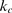 (green), and 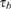 (blue) are varied individually to tune 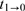 and 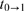 while 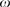 is fixed. The 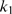 curve (black): 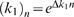, 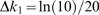; the 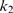 curve (red): 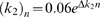, 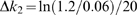; the 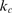 curve (green): 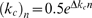, 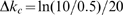; the 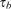 curve (blue): 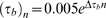, 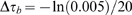. (D) Four sets of kinetic parameters are chosen, and each set corresponds to one curve. On each curve, 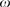 is varied, and the kinetic parameters are fixed. Each point represents an average of 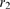 based on 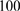 simulations with different noisy signals but fixed 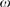. Set 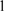 (blue): 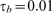, 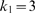, 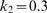. Set 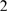 (black): 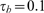, 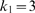, 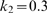. Set 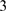 (red): 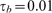, 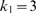, 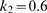. Set 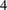 (green): 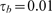, 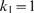, 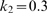. In set 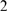, 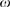 takes 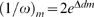, 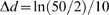. For the rest, 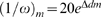, 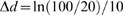. (E) 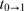 (bottom) and 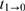 (top) versus 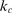. Parameters are the same as the corresponding color set in (D). 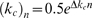, 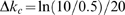. (F) 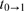 (bottom) and 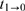 (top) versus 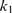. 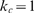 (set 1, blue), 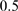 (set 2, red). In each plot, 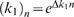, 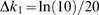. In all simulations, 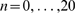, 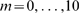, 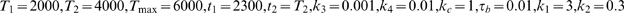, unless otherwise specified.

Once the output of the system reaches the “on” state driven by the stimulus, how does system (1) respond to temporal noises in the input signal 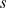
? What are the strategies for effectively maintaining the system in the “on” state even with noises presented by the stimulus? We find that the time scales, denoted by 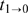 and 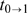 (Figure 2), for the system to switch from the “on” state to the “off” state and from “off” to “on” respectively in the absence of noises in the signal, play a critical role. Specifically, when 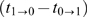 is significantly larger than the time scale of the noise, i.e., 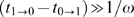, where 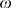 is the frequency of the noise, the output 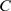 of the system remains in the “on” state (Figures 3A–3B).

Intuitively, when the system in the stable “on” state receives a noisy signal with an instantaneous value possibly near 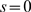, it needs time 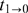 to react and detour to the “off” state. In the case of 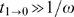, before the system settles down to the “off” state, a noisy signal with an instantaneous value near 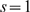 shows up, forcing the system to synchronize with the new value of the input signal. If 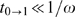, the output recovers fast from the drift towards the inactive state, and is more likely to maintain around the “on” state. The above intuition suggests that the noise attenuation at the “on” state depends positively on 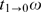 and negatively on 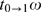. Thus, the quantity 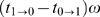, i.e., the signed activation time , could be a good indicator of a system's ability of attenuating noise.

To investigate how noise level in the solution depends on the signed activation time , we study the noise amplification rate, defined as the relative ratio of the coefficients of variation of the output (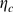) and the noise (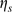) [28]:
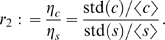
First, we perform numerical simulations on system (1) (Methods) to study the relationship between 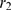 and 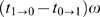 by varying the activation and deactivation time scales while fixing 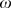. This is achieved by changing the kinetic parameteres 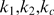, and 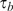 individually in the system, and 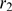 is found decreasing in 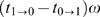 (Figure 3C). Next, we hold 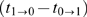 constant, corresponding to no changes in all parameters, and vary the noise frequency 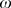. The trend of 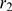 remains the same (Figure 3D). We also consider the dependence of 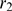 on 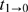 and 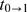 individually (Figure S1). In the single loop case, it turns out that 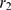 is always decreasing in 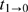 (Figures S1A–S1C), but it might be increasing in 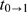 (Figure S1D). Similar results are also obtained for positive-positive-loop systems (Figure S2). Both suggest that neither deactivation nor activation alone can fully characterize the noise amplification rate, and the noise amplification rate is more likely determined by the difference between the deactivation and activation time scales. Next, we further explore this system through the following two analytical approaches.

#### Two-time-scale analysis

To understand why the relative ratio of the time scale of noise in the input and the system's intrinsic time scales when noise is absent is important to noise attenuation, we carry out a two-time-scale asymptotic expansion of the solutions [41]. The solutions are first written in terms of two time scales, 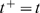 and 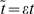, where 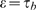,
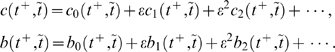
(2)When 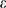 is small, the two time scales are well separated. The independent variables 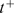 and 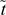 correspond to the fast and slow time scales, respectively. This two-time-scale asymptotic expansion allows us to see a clear dependence of solutions on different time scales. For fast varying input, the input 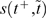 can be written as the sum of a constant signal 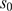 and a fast altering term 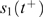, i.e., 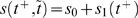. The solution of (1) is (Text S1, Section 4)
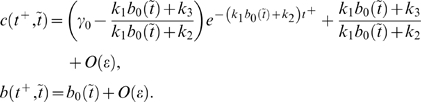
(3)Here, 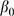 and 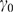 are the initial conditions of 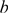 and 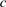, respectively; 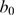 is the solution of
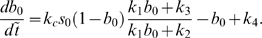
(4)The zero-order solution, 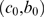, approximates the full solution when 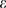 is small (Figures S3A–S3C), and we thus focus on the noise effect on the zero-order solution. Notice that the noise term 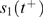 does not show up in equation (4), so the zero-order approximations with and without noise are the same, suggesting that fast varying noises are filtered out through the system (Figures S3D–S3E).

If the input consists of both fast and slow noises (Figures S3G–S3H), for example, when the input is decoupled to a sum of fast and slow noise terms, i.e., 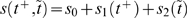, then only the slow part appears in the equation of 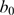 (Text S1, Section 4),
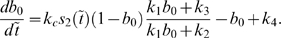
In this case, the noise term 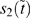 could significantly affect the output (Figures S3F, S3I–S3J). In summary, the single-positive-loop system could function as a low-pass filter [42]–[45], and thus the time scale of the input noise relative to the time scales of the internal system is important to noise attenuation.

#### Fluctuation Dissipation Theorem (FDT) approach

To see the inverse relation between the noise amplification rate and the signed activation time , we employ the FDT approach [27], [28], [46], [47]. Under the linear approximation assumption in FDT, the noise amplification rate can be computed analytically (Text S1, Section 5), and when 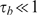 and 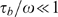, we obtain
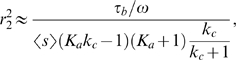
(5)where 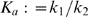 is the association constant, indicating the strength of the activation from 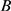 to 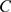.

Based on equation (5), one can infer a qualitative relation between the noise amplification rate and the kinetic parameters. For 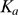, since 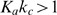 (Text S1, Section 1, the conditions for a “switch-like” response), as 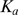 increases, the noise amplification rate 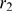 decreases. The parameter 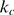, measuring the activation from 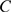 to 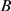, negatively affects the noise amplification rate, and the time scale of the 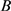-loop, 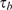, positively affects 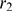.

On the contrary, 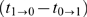 depends negatively on 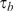 and positively on 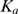 and 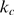 (details later). Thus, a negative relation between 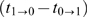 and 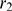 is expected (as confirmed by simulations in Figures 3C–3D). In addition, 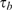 and 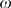 appearing together in (5) suggests a close dependence of the noise attenuation capability on the noise frequency in the input and the intrinsic time scales of the system in the absence of noise.

### How to Control Deactivation and Activation Time Scales

In the previous section, we have demonstrated that the noise amplification rate depends negatively on the signed activation time. Thus, if a system is persistent to noise at the “on” state, it should have a large signed activation time . In this section, by studying the dynamics of the noise-free system, we show that a small 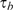 is necessary for a slow deactivation, but not sufficient. With a fixed small 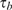, larger 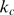 or 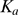 could lead to slower deactivation and faster activation.

#### Deactivation

When the input signal switches from 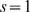 to 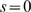, the system responds through deactivation from the stabilized active state to the inactive state. The dynamics of 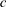 around the inactive state can be approximated by (Text S1, Section 1)
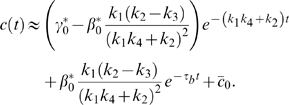
(6)Here, 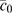 and 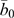 denote the steady state values of 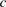 and 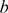 at 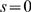, respectively; 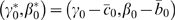, where 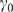 and 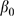 are the initial conditions of 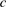 and 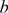, respectively. Equation (6) clearly indicates that the dynamics of 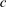 is not only determined by parameters in the 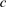-equation but also affected by the time scale of the 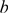-equation, 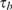. Without the positive feedback loop (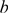-equation), 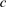 can be solved in a closed form:
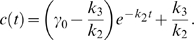
(7)Comparing (7) with (6), it is clear that, to achieve a slower deactivation when 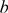 is present, 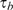 need to be much smaller than 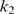. Conversely, if 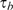 is on the same or higher order of 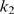, the output 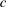 responds in a time scale of 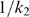 without slow deactivation.

In addition to a small 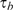, the more significantly the second term in (6) contributes to the dynamics of 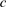, the slower 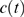 converges to 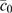, and thus, the larger 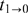 becomes. The contribution of 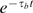 to the deactivation time scale is characterized by (Text S1, Section 1)
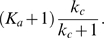
(8)As a result, a large 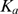 or 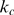 leads to a slow deactivation. This is also demonstrated by direct simulations (Figures 3E–3F, top).

In addition to the linear stability analysis around either the active state or the inactive state, under the assumption of 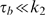, our previous two-time-scale asymptotic expansion provides a uniformly approximated solution of (1) [41]. The leading order of 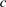 yields a solution that is in a similar form of (6) (Text S1, Section 4):
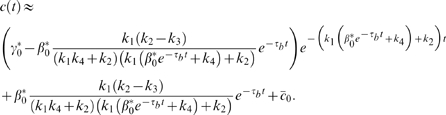
(9)


#### Activation

Besides the deactivation time scale, the slow positive loop 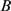 also affects the activation time scale. During the activation process (Text S1, Section 1),
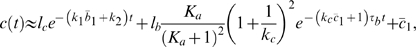
(10)where 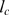 and 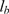 are two constants depending on the initial conditions; 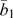 and 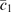 denote the steady state of 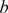 and 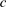 at 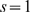, respectively. Different from the deactivation process, loop 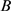 affects the dynamics of 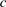 through the term
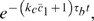
(11)instead of 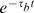. The extra factor 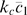 in the exponent of (11) can lead to faster activation. Numerical simulations of system (1) with different values of 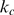 confirm this (Figure 3E, bottom). Another way to accelerate the activation process is to minimize the contribution of the exponential function in (11) to the dynamics of 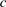, characterized by (Text S1, Section 1)
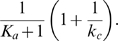
(12)Based on (12), increasing 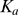 or 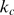 decreases the contribution from 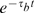, and thus leads to faster activation (Figures 3E–3F, bottom). In the extreme case of 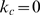, there is no feedback from the output 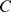 to the 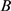 system, and 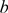 evolves on its own time scale of 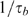. Thus, the output 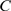 driven by 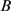 is also on the slow time scale of 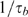.

In summary, a slow positive feedback loop is necessary for slow deactivation, and a slow positive feedback can lead to fast activation. It is worth pointing out that the above analysis of achieving rapid activation and slow deactivation is based on a system with one positive feedback loop. In a previous study [34], the response of rapid activation and slow deactivation was achieved through two positive feedback loops with two drastically different time scales. This raises the question of why biological processes often utilize multiple loops rather than a single positive feedback loop when one positive feedback loop seems sufficient for the basic objective.

### Roles of an Additional Positive Feedback Loop: Faster Activation and Robustness

In many biological processes, such as cell cycle [5], [6], often two positive feedback loops 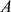 and 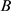 activate the output 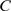 simultaneously (Figure 1A). Similar to system (1), the corresponding equations take the form:
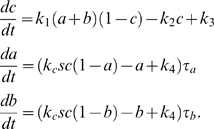
(13)


**Figure 4:**
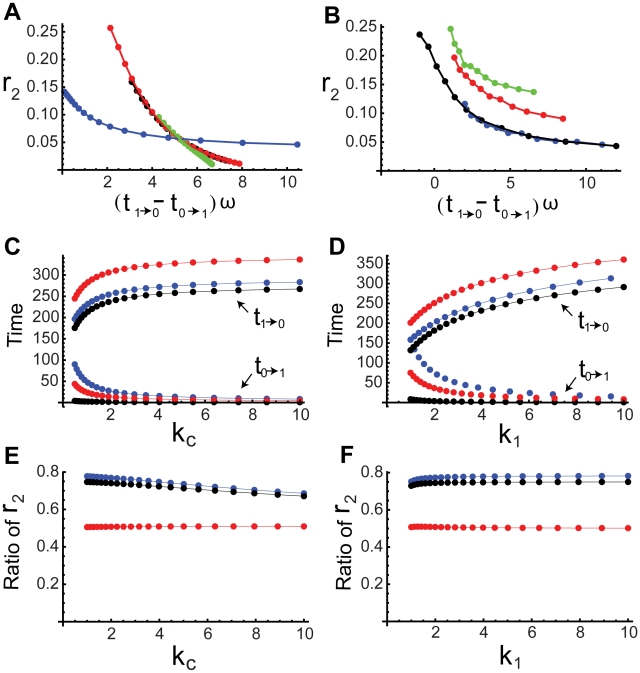
Noise attenuation and time scales in positive-positive-loop systems. (A–B) The same plots as in Figures 3C–3D but with the additional positive feedback loop 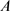, where 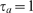. (C–D) The change of 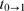 (bottom) and 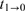 (top) with respect to 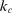 (C) and 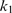 (D) in single-positive-loop (blue), fast-slow-loop (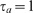, black), and slow-slow-loop (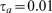, red) systems. 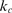 and 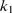 are varied the same way as in Figure 3E and Figure 3F, respectively. (E–F) The ratio of 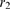 in positive-positive-loop systems to 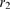 in the corresponding single-positive-loop systems with respect to 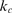 (E) and 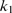 (F). 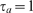 (blue), 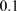 (black), and 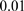 (red). All simulations use the same parameters and inputs as their counterparts in Figure 3 with the additional parameter 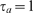, unless otherwise specified.

Through direct numerical simulations, we find that the noise amplification rate decreases in the signed activation time (Figures 4A–4B), following the same principle as in the single-positive-loop system (1). The activation time scale decreases in 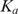 and 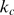, while the deactivation time scale increases in 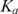 and 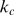 (Figures 4C–4D, Table 1). We also find that an additional feedback loop can lead to a faster activation (red and black versus blue in Figures 4C–4D, bottom) and a slower (or similar) deactivation (red and black versus blue in Figures 4C–4D, top), compared to a single-positive-loop system, and a positive-positive-loop system can achieve similar activation and deactivation rates with larger ranges of kinetic parameters than a single-positive-loop system (Table 2). Consequently, noise attenuation can be better achieved in the positive-positive-loop system (Figures 4E–4F). Below are details of the mathematical analysis for the roles of the additional positive feedback.

**Table 1:** Qualitative relationship between response time scales and parameters 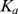, 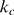, 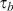, and 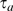 in single-positive-loop (S-P), positive-positive-loop (P-P), positive-negative-loop (P-N) systems.

	Deactivation			Activation		
	S-P	P-P	P-N	S-P	P-P	P-N
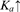	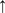	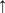	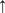	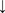	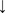	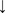
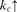	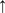	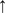	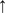	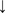	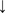	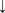
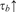	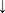	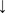	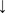	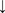	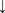	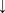
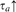	NA	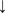	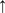	NA	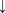	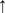

The up arrow 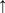 and down arrow 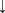 denote increasing and decreasing, respectively. Variables 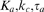, and 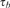 are positive.

**Table 2:** Changes of the activation and deactivation time scales with respect to parameter variations in single-positive-loop (S-P), fast-slow-loop (F-S), and slow-slow-loop (S-S) systems.

Parameter	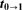	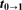	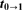	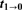	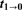	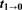
changes	S-P	F-S	S-S	S-P	F-S	S-S
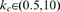	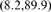	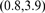	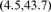	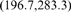	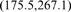	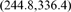
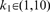	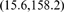	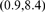	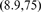	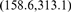	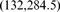	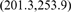
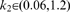	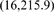	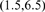	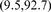	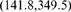	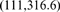	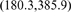

For example, when the parameter 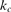 varies between 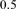 and 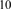, the activation time scale in the S-P system varies between 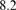 and 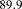; the activation time scale in the F-S system (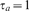) varies between 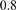 and 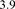; the activation time scale in the S-S system (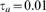) varies between 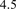 and 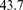.

#### Activation

During activation, the dynamics of 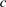 can be approximated by (Text S1, Section 2)
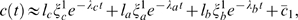
(14)where 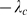, 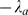, and 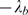 denote the eigenvalues of the Jacobian matrix of system (13) at the active state; 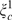, 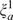, and 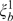 are the first coordinates of the corresponding eigenvectors, respectively; 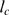, 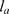, and 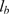 are constants depending on initial conditions. Similar to the single-positive-loop case, in order to achieve slow deactivation, either 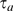 or 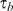 must be much smaller than 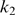. Without loss of generality, we assume that 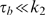 and 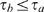 because of the symmetry between the two loops. Thus, analytically, we consider the following two cases to illustrate the effect of the additional feedback loop.

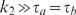
, corresponding to a slow-slow-loop system. Loop 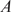 and loop 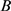 both affect the dynamics of 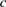 through the term 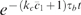, and their contributions are measured by (Text S1, Section 2)
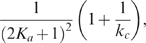
(15)which is smaller than (12), the corresponding contribution of loop 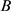 to the single-positive-loop system. If 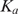 is large compared to one, even though the additional loop is on the slow time scale, the activation time scale can drop to one quarter of that in the single-positive-loop system, which is also suggested by direct simulations (Figures 4C–4D, lower red dots).

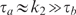
, corresponding to a fast-slow-loop system. In this case, the term 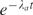 decays much faster than 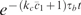. As a result, the slow dynamics of 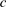 mostly comes from loop 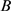 through the term 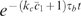. The contribution from 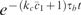 is characterized by (Text S1, Section 2)
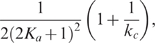
(16)which is also smaller than (12). Notice that, if 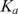 is large compared to one, (16) can be as small as one eighth of (12). Direct numerical simulations also show that a fast-slow-loop system has much smaller 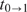 than the corresponding single-positive-loop system (Figures 4C–4D, lower black versus blue).

In summary, both cases suggest that an additional positive feedback loop accelerates the activation process, and the activation time scale decreases in 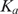 and 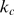 (Figures 4C–4D, bottom), similar to the single-positive-loop system.

#### Deactivation

During deactivation, the dynamics of 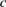 is approximated by (Text S1, Section 2)
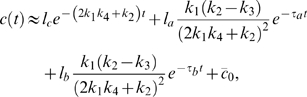
(17)where 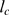, 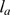, and 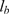 are constants depending on initial conditions of the system; 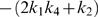, 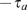, and 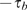 are the eigenvalues of the Jacobian matrix of system (13) at the inactive state. Let us consider the same two cases studied in the activation process.

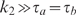
, corresponding to a slow-slow-loop system. The contributions of loop 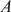 and loop 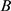 to the dynamics of 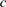 are measured by (Text S1, Section 2)
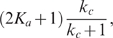
(18)which is larger than (8), the corresponding contribution of loop 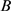 to a single loop system. So, the additional slow positive feedback loop sustains the deactivation process, as is also shown in direct simulations (Figures 4C–4D, upper red dots).

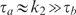
, corresponding to a fast-slow-loop system. In this case, the contribution of the term 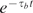 to the dynamics of 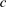 is (Text S1, Section 2)
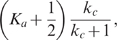
(19)smaller than (8). In other words, the deactivation time scale in a positive-positive-loop system can be faster than that in a single-positive-loop system. However, the relative difference of the deactivation time scales between the two systems is small (Figures 4C–4D, upper black and blue dots), because the ratio of (19) to (8) is 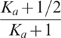

The above analysis suggests that the additional loop 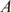 increases the deactivation time in a slow-slow-loop system and slightly decreases the deactivation time in a fast-slow-loop system. Equations (18) and (19) also suggest the positive dependence of the deactivation time scale on 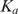 and 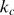, as confirmed by direct simulations (Figures 4C–4D, top).

Moreover, as seen in Table 2, the activation time scale, 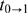, is under tighter control in a positive-positive-loop system than a single-positive-loop system when the kinetic parameters are varied. In other words, a change of the kinetic parameters in a positive-positive-loop system leads to less change in the activation time scale than in a single-positive-loop system (Table 2); therefore, the activation time scale in a positive-positive-loop system is more robust to fluctuations in kinetic parameters (independent of fluctuations in the input).

Even though the additional loop can lead to a slightly larger deactivation time under certain conditions (e.g., 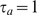), the relative change is usually small, especially in comparison to the relative decrease of the activation time (Table 2). As a result, 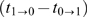 increases, and thus the noise amplification rate becomes smaller in the positive-positive-loop system than in the corresponding single-positive-loop system (Figures 4E–4F, blue dots). Of course, when the additional loop 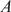 is slow (e.g., 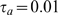), the deactivation time scale increases, and the activation time scale decreases, resulting in better noise attenuation than the single-positive-loop system (Figures 4E–4F, red dots).

### Roles of an Additional Negative Feedback Loop: Slower Deactivation

In this section, we study how an additional negative feedback loop affects noise attenuation in a system. One of the simplest ways to introduce negative feedback to the single-positive-loop system (1) is to let 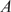 deactivate 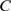 (Figure 1B) [21]. In this case, the model becomes
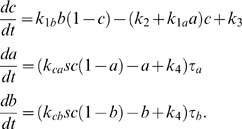
(20)


**Figure 5:**
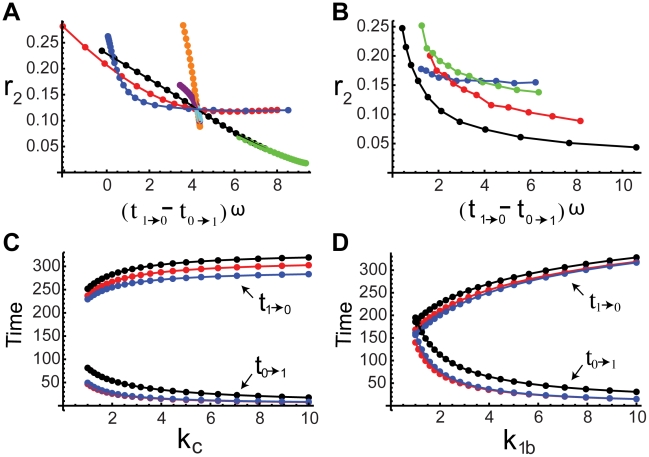
Noise attenuation and time scales in positive-negative-loop systems. (A) Kinetic parameters 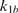 (black), 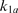 (orange), 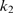 (red), 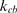 (green), 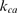 (purple), 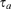 (cyan), and 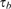 (blue) are varied individually to tune 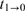 and 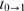 while 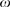 is fixed. In each parameter variation, 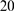 samples are simulated. (B) The dependence of 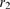 on 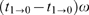 when 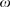 is varied and the kinetic parameters are fixed. We use the same four sets of parameters as in Figure 3D with the additional parameters 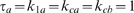. (C–D) The change of 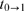 (bottom) and 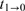 (top) with respect to 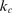 (C) and 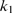 (D) in single-positive-loop (blue), positive-negative-loop (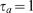, black), and positive-negative-loop (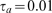, red) systems. 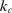 and 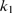 are varied the same way as in Figure 3E and Figure 3F, respectively.

Our analytical results show that the additional negative feedback loop leads to slower deactivation and slower (or slightly faster) activation compared to its single-positive-loop counterpart (red and black versus blue in Figures 5C–5D). Moreover, the deactivation time scale increases in 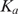 and 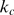, and the activation time scale decreases in 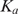 and (Figures 5C–5D, Table 1), similar to the single-positive-loop (Figures 3E–3F) and positive-positive-loop systems (Figures 4C–4D). Numerical simulations reinforce these findings and demonstrate that the noise amplification rate of negative-positive-loop systems decreases in the signed activation time , following the same principle as their single-positive-loop counterparts (Figures 5A–5B).

Below, we provide detailed analysis to show how the deactivation and activation time scales depend on various kinetic parameters, compared to the single-positive-loop case. In our analytical studies, we assume 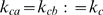 for simplicity. However, 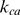 and 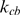 are varied independently in numerical simulations.

#### Deactivation

During deactivation, the dynamics of 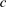 is approximated by (Text S1, Section 3)
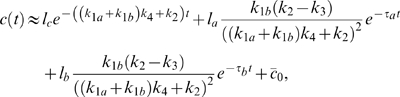
(21)where 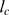, 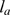, and 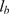 are constants depending on initial conditions of the system; 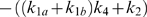, 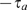, and 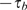 are the eigenvalues of the Jacobian matrix of system (20) at the inactive state. We focus on the following two cases.

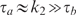
: fast negative loop and slow positive loop. In this case, the contribution of 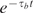 to the dynamics of 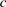 is measured by (Text S1, Section 3)
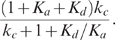
(22)Here, 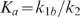, the same as in the single-positive-loop case; 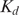 is defined as 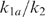. A straightforward calculation shows that (22) is always larger than (8), the corresponding contribution of 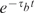 to the single-positive-loop system (Text S1, Section 3). Note that the more the 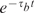 term contributes to the dynamics, the slower 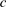 gets deactivated. As a result, the deactivation in the fast-negative-slow-positive-loop system is slower than that in the single-positive-loop system. In addition, (22) increases in 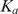 and 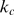, and thus the deactivation time scale increases in 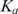 and 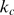.

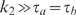
: slow negative loop and slow positive loop. The contribution from the slow term 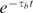 is measured by (22) minus a small term on the order of 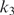 and 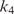 (Text S1, Section 3), and is still larger than (8).

To summarize, in both cases the additional negative feedback loop leads to slower deactivation, and the deactivation time scale increases in 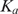 and 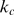 (Figures 5C–5D, top).

#### Activation

We again analyze the two cases of fast negative loop and slow negative loop.

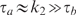
: fast negative loop and slow positive loop. The slow dynamics of 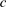 is characterized by (Text S1, Section 3)
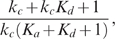
(23)which is bigger than (12), the corresponding contribution to the single-positive-loop system. In addition, (23) is decreasing in 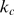 and 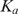.

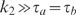
: slow negative loop and slow positive loop. The contribution to the slow dynamics of 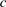 is measured by (Text S1, Section 3)
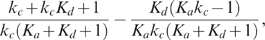
(24)which is smaller than (12). The function (24) decreases in 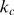 and 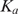.

Together, compared to the single-positive-loop system, the activation is slower when the negative feedback acts on a fast time scale (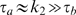), but faster when the the negative feedback is on a slow time scale (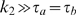). Numerical simulations confirm these findings (Figures 5C–5D), and show that in the slow negative loop case, the activation is about the same as the single-positive-loop case, albeit slightly faster (Figures 5C–5D, lower red versus blue). Moreover, the activation time scale decreases in 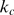 and 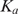 (Figures 5C–5D, lower).

Since the additional negative feedback loop in general leads to slower deactivation and slower activation, the net effect to the signed activation time is not straightforward. Numerical simulations suggest that the noise amplification rate could either increase or decrease depending on 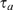, the time scale of the negative feedback loop (Figure S4).

### Noise Attenuation in a Yeast Cell Polarization System

**Figure 6:**
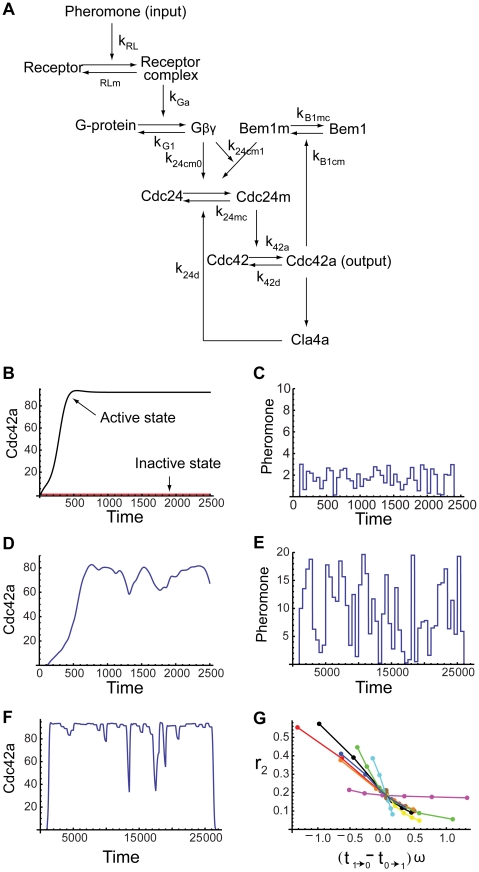
Noise attenuation in a yeast cell polarization model. (A) Schematic diagram of the yeast cell polarization signal transduction pathway. (B) The active state (upper black) and the inactive state (lower red). The upper black curve is the output (concentration of Cdc42a) response to the constant high pheromone concentration of [L]
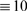
nM, and the lower red curve is the output response to the low pheromone concentration of [L]
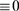
nM. (C) A noisy input signal with low amplitude. (D) The output response to (C). (E) A noisy input signal with large amplitude. (F) The output response to (E). (G) The noise amplification rate versus the signed activation time. Ten parameters are varied systematically in 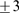-fold ranges based on their original values given in (D). Each variation corresponds to one curve on the plot. The ten parameters are 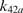 (red), 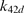 (black), 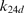 (pink), 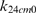 (magenta), 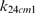 (yellow), 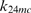 (orange), 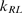 (cyan), 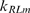 (green), 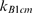 (blue), 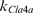 (brown). The leftmost point of the 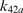 curve is not shown in this picture, as it changes the scale of the picture. Please see Figure S7 for the full plot. Parameter values are mostly taken from [37], except 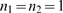 and 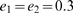, because of the loss of the spatial effect. The initial conditions are 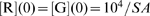, 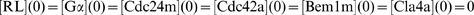, where 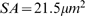.

Unlike the simple models in the previous section, a yeast cell polarization signaling pathway model that we study next (Figure 6A) consists of more than three components and multiple feedback regulations [37], [48]. Polarization in yeast cells (**a** or 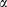 cells) is activated by pheromone gradients [48]. The pheromone (L) binds to the receptor (R) and becomes activated (RL). The activated receptor facilitates the conversion of the heterotrimeric G-protein (G) into an activated 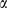-subunit (G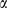) and a free G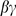 dimmer [49]. G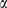 is then deactivated to an inactive 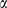-subunit (Gd), which in turn binds to G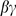 and forms the heterotrimeric G-protein. The free G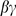 recruits cytoplasmic Cdc24 to the membrane, forming the membrane-bounded Cdc24 (Cdc24m), an activator of Cdc42. Accumulation of the activated Cdc42 (Cdc42a) at the projection site is a key feature of polarization, and thus is regarded as the output of the proposed system. The activated Cdc42 participates in other polarization processes, forming positive or negative feedback loops. For example, the activated Cdc42 sequesters the scaffold protein Bem1 to the membrane, which then recruits Cdc24 to the membrane [50]. This forms a positive feedback loop. Other functions of Cdc42 include the activation of Cla4 (Cla4a), an inhibitor of Cdc24, resulting in a negative feedback loop [51].

Following the model proposed in [37] but ignoring the spatial effect, we have the following system of equations:
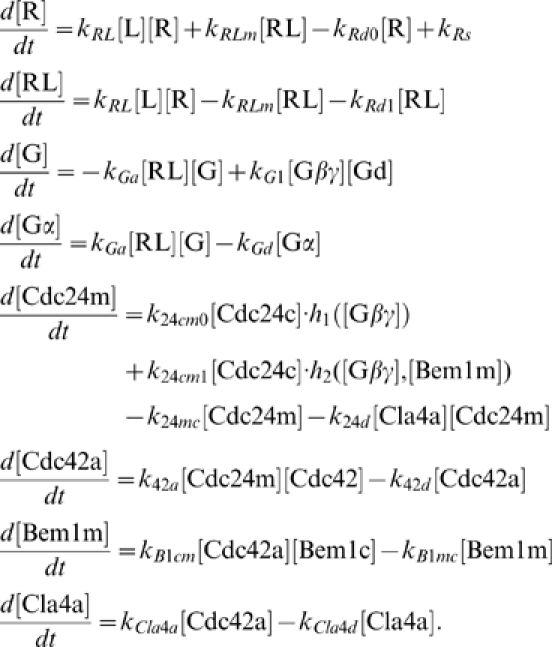
(25)Here, 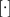 denotes the concentration of the corresponding protein; [L] is the input signal, and [Cdc42a] is the output; the concentrations of G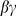, Gd, the inactive form of Cdc42, the cytoplasmic Cdc24, and the cytoplasmic Bem1 are derived through conservation relations:
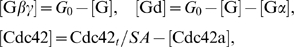


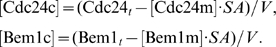
Here, 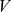 is the volume of the cell; 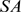 is the surface area of the cell; 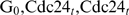, and 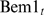 are the total numbers of molecules per cell of the corresponding proteins. The two Hill functions 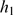 and 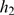 are defined as
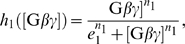


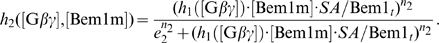
These two functions represent two different ways of bringing Cdc24 to the membrane. One is by the free G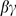 (function 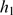), and the other is through Bem1. The Bem1 recruitment is known to be facilitated by G
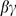
's binding to Ste20 [52], and the influence from G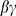 is modeled by the function 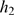. Kinetic parameters take the same values as in [37], and see also the caption of Figure 6.

Starting from zero Cdc42a, giving high ([L]
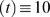
nM) or low ([L]
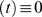
nM) constant inputs, the output reaches active and inactive states, respectively, which are clearly distinguished (Figure 6B). Inputs with small amplitude (Figure 6C) can be detected by the system (Figure 6D). On the other hand, the output is robust to noise when it is around the active state (Figures 6E–6F). To study how the noise amplification rate depends on the relative time scales, we vary ten parameters systematically in their 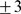-fold ranges. All of them show the same decreasing trend of the noise amplification rate as a function of the signed activation time (Figure 6G). This suggests that the negative relation between the noise amplification rate and the signed activation time , derived from the simple models, could also apply to models of complex interactions and combinations of positive and negative feedback loops. Such negative relationship may be a generic principle on noise suppression for input-output systems with feedback loops.

### Applications to Other Systems

#### A polymyxin B resistance model in enteric bacteria

**Figure 7:**
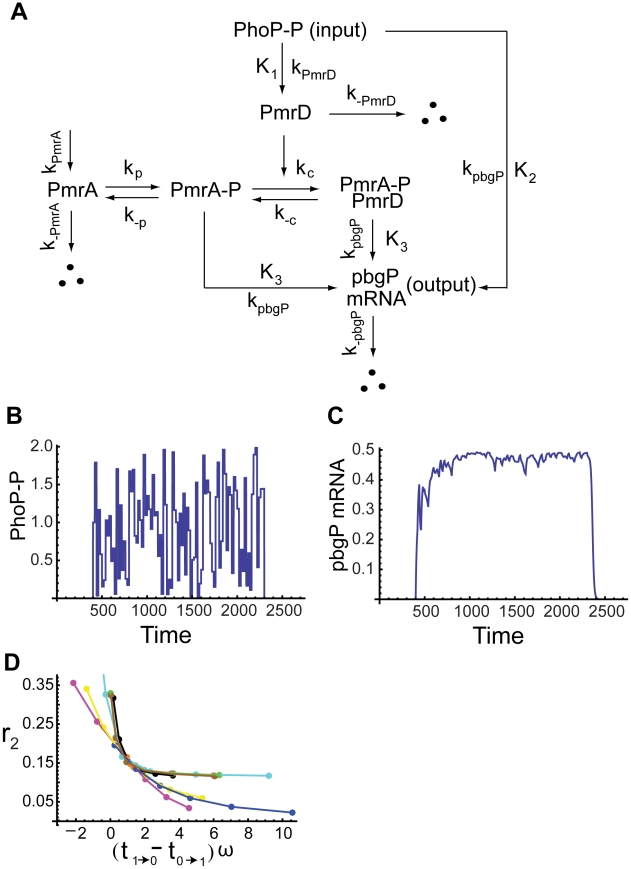
Noise attenuation in a polymyxin B resistance model. (A) Schematic diagram of the polymyxin B resistance network. (B) A typical input with noise. (C) The output response to the input in (B). (D) The noise amplification rate versus the signed activation time . Ten parameters are varied in 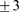-fold ranges based on their original values given in Table S1. The ten parameters are 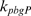 (red), 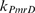 (black), 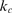 (pink), 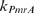 (magenta), 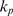 (yellow), 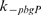 (orange), 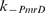 (cyan), 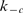 (green), 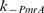 (blue), 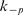 (brown). The equations of the system are given in Section 7 of Text S1.

To further explore the generality of the proposed criterion, we consider a recently discovered genetic regulatory network of the connector-mediated polymyxin B resistance induced by 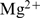 in enteric bacteria [38]. At low 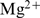, the protein PhoP is phosphorylated and activates the promoter of the connector protein PmrD. PmrD then proceeds to activate the transcription factor of pbgP, which eventually results in the resistance to polymyxin B. In addition to the indirect regulation, PhoP also promotes pbgP expression directly by binding to the pbgP promoter [38]. The feedforward connector loop (FCL) model proposed in [38] contains five variables and 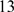 parameters with the input being the concentration of the phosphorylated PhoP and the output being the pbgP mRNA level (Figure 7A).

Interestingly, the FCL model robustly exhibits fast activation and slow deactivation as shown in [38], which would lead to a strong noise attenuation capability based on our proposed criterion. Indeed, when noise is introduced to the input (Figure 7B), our simulation shows the output, pbgP mRNA, maintains at a high level (Figure 7C). The noise amplification rate is found to decrease as the signed activation time increases (Figure 7D) when ten out of thirteen parameters in the model are varied within their 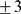-fold ranges. (Please see Section 7 of Text S1 for the equations and Table S1 for the parameter values.)

#### Four connector-mediated models

Following the work [38], Mitrophanov and Groisman proposed four different regulatory mechanisms of a connector-mediated circuit [39]. The four generic models, mainly consisting of three components, the connector, the sensor, and the regulator, differ in functions of the connector protein. In the regulator-protecting (RP) model, the connector protein binds to the phosphorylated regulator and protects it from dephosphorylation by the sensor protein, whereas in the regulator-activating (RA) model, the connector binds to the unphosphorylated regulator and promotes its phosphorylation. The connector in the phosphatase-inhibiting (PI) model binds to the sensor to inhibit its phosphatase activity, instead of promoting the kinase activity as in the kinase-stimulating (KS) model. The same input used for the four models is the synthesis rate of the connector protein, and their output is the concentration of the phosphorylated regulator protein [39].

**Figure 8:**
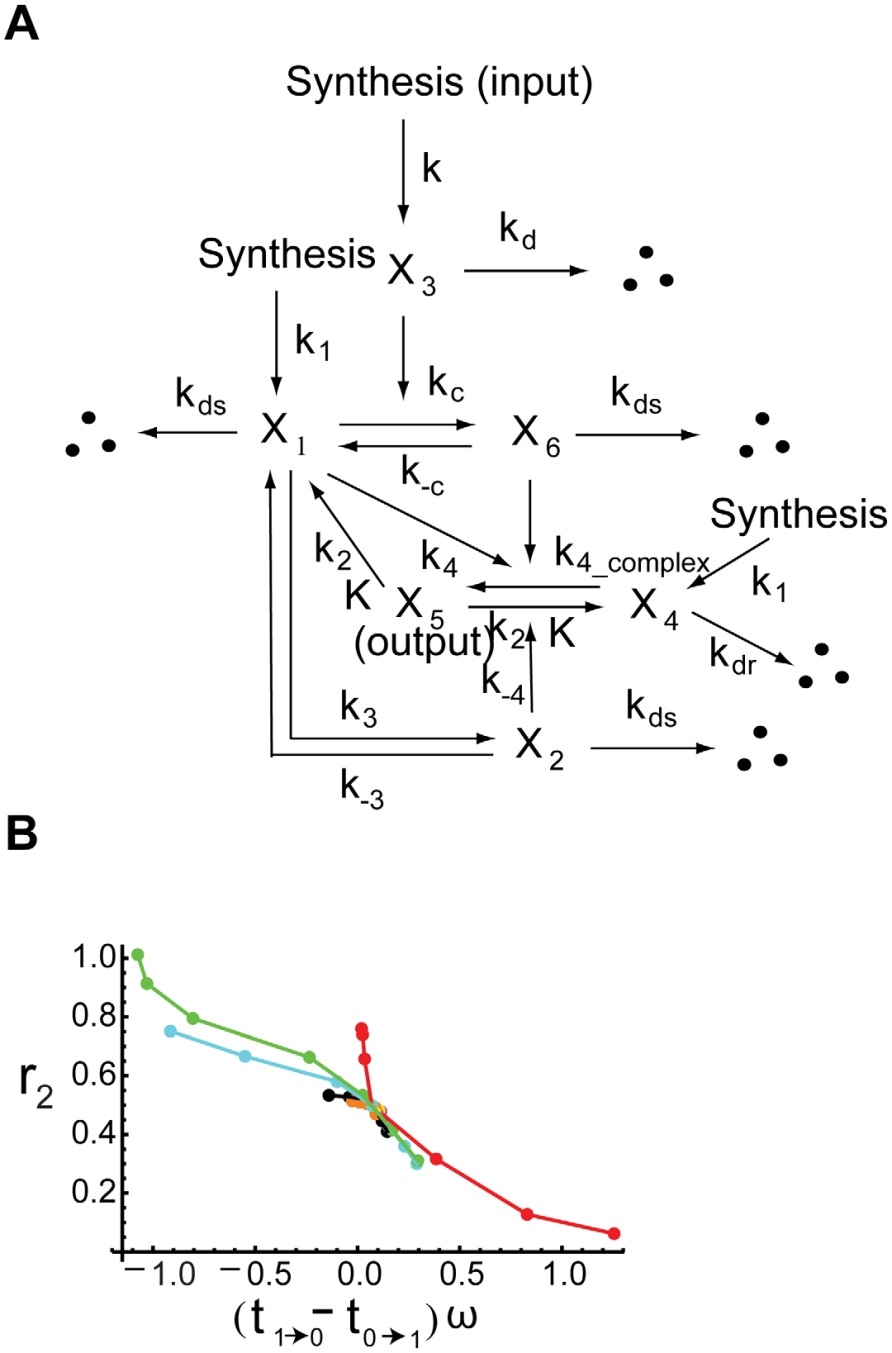
Noise attenuation in the kinase-stimulating (KS) model. (A) Schematic diagram of the KS network. Here, 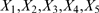, and 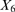 represent the sensor protein in the kinase form, the sensor protein in the phosphatase form, the connector protein, the response regulator, the phosphorylated response regulator, and the connector-sensor(kinase) complex, respectively. (B) The noise amplification rate versus the signed activation time . Eight parameters are varied in 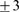-fold ranges around their original values given in [39]. The eight parameters are 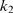 (red), 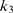 (black), 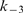 (pink), 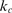 (magenta), 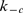 (yellow), 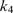 (orange), 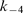 (cyan), and 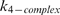 (green).

We first study the KS model (Figure 8A) to test the relation between the noise amplification rate and the signed activation time. The KS model used in [39] contains six variables and 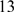 parameters. Based on the same parameter set used in [39], we vary eight parameters within their 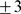-fold ranges individually. The simulations consistently indicate the inverse relationship between the noise amplification rate and the signed activation time (Figure 8B), similar to our results for other systems.

Next, we study the four models and compare their noise amplification rates and signed activation time s for the same nominal parameter set used in [39]. Although the deactivation and activation dynamics of the four models are quite different [39] (Figures S8A–S8B), we notice that the same relationship between the amplification rate and signed activation time seems to hold across the four different models, i.e. a model with smaller signed activation time has higher noise amplification rate than another system with larger signed activation time (Table 3, Figures S8C–S8F).

**Table 3:** Time scales and noise amplification rates in four connector-mediated models.

	RP	RA	KS	PI
Activation time ( 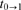 )	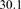	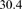	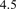	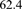
Deactivation time ( 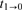 )	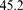	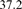	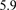	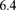
Signed activation time	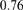	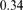	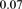	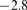
Noise amplification rate	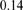	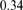	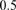	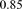

RP, RA, PI, and KS stands for the regulator-protecting model, the regulator-activating model, the phosphatase-inhibiting model, and the kinase-stimulating model, respectively. The same noise input (Figure S8G) is used for all four models. The equations and parameters are taken from [39].

## Discussion

Our theoretical and numerical studies have demonstrated that it is not the sign of the feedback that determines the degree of noise attenuation. In searching for a general framework for a relation between feedback and noise attenuation, we have identified a critical quantity, termed as the “signed activation time”. Its relation with the system's ability of noise attenuation has been explored, and we have revealed that the noise amplification rate decreases in the signed activation time. These results are concluded through employing multiple time scale analysis, Fluctuation Dissipation Theorem, and linear stability analysis, combined with numerical simulations, in three feedback modules (Figure 1): single-positive-loop, positive-positive-loop, and positive-negative-loop systems. To test the generality of the conclusion, we have explored models (Figure 1A) with saturation effect, i.e., modeling feedback loops by Hill functions (Text S1, Section 6, Figures S5, S6), a yeast cell polarization model consisting of multiple intermediate components (Figure 6), a polymyxin B resistance model in enteric bacteria (Figure 7), and four connector mediated models (Table 3, Figure 8). In all cases, the noise amplification rate has been confirmed to be a decreasing function in the signed activation time.

To analyze the roles of multiple positive and negative feedback loops in our toy models, we have found that: 1) an additional positive feedback loop could drastically reduce the activation time scale, improving performance in noise attenuation; 2) the time scales in positive-positive-loop feedback systems are more robust to rate constant variations (e.g. due to variability of organisms or variation of environments); and 3) adding a negative feedback loop usually sustains both deactivation and activation processes, and thus its overall effect on the signed activation time could be either negative or positive.

To obtain slow deactivation and fast activation, we have identified two key parameters, 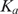, the association constant of 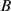 to 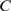, and 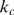, the association constant of 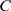 to 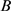 (Figure 1A), that tightly control the deactivation and the activation time scales (Tables 1 and 2). Interestingly, under appropriate conditions, even the simplest *single* positive feedback loop system could display slow deactivation and fast activation, which were not observed in previous works [34]–[36].

The idea of connecting noise attenuation with the time scales of signal responses was mentioned in other works, for example, [53], in which only the activation time scale was considered. However, we have shown that in our models neither the deactivation time scale nor activation time scale alone predict correctly the trend of the noise amplification rate (comparing Figure 3C to Figures S1C–S1D, for example) and the noise amplification rate is an interplay between the two time scales. Our proposed quantity, the signed activation time , provides a more consistent relation linking to the noise attenuation rate.

Direct approaches for analyzing noise may be applied to feedback systems, such as the energy landscape method [4], [35], [54]–[56] and the methods used for noise attenuation or amplification in signaling cascades [28], [57]–[60] and covalent modification cycles [53]. To characterize signaling time scales, we have studied the magnitude of eigenvalues and their corresponding eigenvectors of the Jacobian matrices at each distinct state of the signal. Questions concerning how the magnitude of signal output and signal duration depend on properties of pathway components (e.g., the effect of cascades) were explored from a system control point of view in other works [61]–[63].

Our study features a novel approach using multiple time scale asymptotic expansion [41]. Different from the one-time-scale expansion, this approach provides an explicit relation between the solutions and the two separated time scales, suggesting that the single-positive-loop system can function as a low-pass filter and explaining why the relative size of noise time scale and a system's intrinsic time scales is important to noise attenuation. This approach may be applied to other biological systems with time scale separations.

Our findings suggest that the negative relationship between the noise amplification rate and the signed activation time could be a general principle for many biological systems regardless of specific regulations or feedback loops. Notice that the deactivation and activation time scales are widely defined and could be measured without detailed knowledge of a system's internal structure. Thus, the underline system could be treated as a black box and its ability of noise attenuation could be estimated based on the signed activation time . In general, if a system prefers to better attenuate noise at the “on” state, the system should have a large signed activation time .

We would like to point out that the studies done here mainly focus on time scale changes within a fixed system, although comparisons across different systems are likely to be consistent with our result (e.g. the four connector-mediated models). However, we might not expect two drastically different systems with equal signed activation time to exhibit the same noise amplification rate, which is likely to depend on other factors in the system as well. We hope that the present work can shed some light on general principles of noise attenuation, in particular, their connections with timing of a system in the absence of noises.

## Methods

### Simulations

All simulations are performed using Mathematica 6.0.0. To compute the noise amplification rate 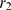, we use
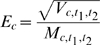
to approximate 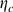, where
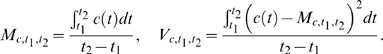
We use
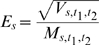
to approximate 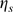, where
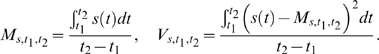
The noise is generated by dividing the time interval into sub-intervals of length 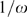, and on each sub-interval the signal takes a random number from a uniform distribution in 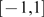. See Figure 3A for a typical noisy signal.

### Linear analysis, two-time-scale asymptotical expansion, FDT approach

See Text S1.

## Supporting Information

Text S1 Linear analysis, two-time-scale asymptotical expansion, FDT approach, and equations of the polymyxin B resistance model.
(0.18 MB PDF)

Figure S1 Noise amplification rate in single-positive-loop systems with respect to *t_1→0_* and *t_0→1_*, respectively.
(0.08 MB PDF)

Figure S2 Noise amplification rate in positive-positive-loop systems with respect to *t_1→0_* and *t_0→1_*, respectively.
(0.07 MB PDF)

Figure S3 Two-time-scale decomposition of the single-positive-loop system (1) in the main text.
(0.23 MB PDF)

Figure S4 The ratio of noise amplification rates in positive-negative-loop systems to single-positive-loop systems.
(0.03 MB PDF)

Figure S5 Simulations of the Hill function model (46) in Text S1.
(0.10 MB PDF)

Figure S6 Simulations of the Hill function model (47) in Text S1.
(0.07 MB PDF)

Figure S7 The full plot of Figure 6G.
(0.03 MB PDF)

Figure S8 Simulations of the four connector-mediated models. The activation (A) and deactivation (B) dynamics of the regulator-protecting (RP) model (blue), the regulator-activating (RA) model (green), the phosphatase-inhibiting (PI) model (black), and the kinase-stimulating (KS) model (red). (C–F) The output of the RP model (C), the RA model (D), the PI model (E), and the KS model (F). In (C–F), we use the same input (G).
(0.09 MB PDF)

Table S1 Parameters used in the simulation of the polymyxin B resistance model. The values of *k_p_* and *k_−p_* correspond to *k_p_^max^ = 1*, *k_−p_^max^ = 2*, and *f = 0.05* in [38], a case of mild activation from the second input.
(0.05 MB PDF)
