# Supplementary material for: A Critical Quantity for Noise Attenuation in Feedback Systems
Source: PLoS Comput Biol. 2010 Apr 29;6(4):e1000764. doi: 10.1371/journal.pcbi.1000764 (PMC2861702; doi:10.1371/journal.pcbi.1000764)
Supplement: Text S1 — Linear analysis, two-time-scale asymptotical expansion, FDT approach, and equations of the polymyxin B resistance model. (0.18 MB PDF) [file pcbi.1000764.s001.pdf]

Text Supplementary 1 —— A Critical Quantity for Noise Attenuation in Feedback Systems

Liming Wang, Jack Xin, Qing Nie

Center for Mathematical and Computational Biology, Center for Complex Biological Systems, and Department of Mathematics, University of California at Irvine, Irvine, CA 92697, United States of America

∗ E-mail: qnie@math.uci.edu

1 Single-Positive-Loop Module

The following ordinary differential equations can be used to describe the dynamics of a single-positive-loop module (inside the red dashed box of Figure 1A):

$$\begin{aligned}\frac{dc^*}{dt} &= k_1^* b^* (c_{\text{tot}} - c^*) - k_2^* c^* + k_3^*, \\ \frac{db^*}{dt} &= (k_c^* s^* c^* (b_{\text{tot}} - b^*) - k_5^* b^* + k_4^*)\tau_b^*.\end{aligned} \tag{1}$$

Here, c^* and b^* are the active levels of the corresponding components, varying between $[0, c_{\text{tot}}]$ and $[0, b_{\text{tot}}]$, respectively. The variable s^* stands for the input, whose maximum is s_{max}. For the convenience of analysis, we assume that the total amount of the active form and inactive form are conserved in each component, as in previous works [2, 13]. Later on, we test our conclusion in other models with degradation/dilution terms (e.g. a yeast cell polarization model and a polymyxin B resistance model in enteric bacteria).

Next, we rescale the variables by defining

$$c = \frac{c^*}{c_{\text{tot}}}, \quad b = \frac{b^*}{b_{\text{tot}}}, \quad s = \frac{s^*}{s_{\text{max}}}.$$

Now, equations in (1) becomes

$$\frac{dc}{dt} = k_1^* b_{\text{tot}} b(1-c) - k_2^* c + \frac{k_3^*}{c_{\text{tot}}}, \tag{2}$$
$$\frac{db}{dt} = (\frac{k_c^*}{k_5^*} s_{\text{max}} c_{\text{tot}} sc(1-b) - b + \frac{k_4^*}{k_5^* b_{\text{tot}}})\tau_b^* k_5^*.$$

Let

$$k_1 = k_1^* b_{\text{tot}}, \quad k_2 = k_2^*, \quad k_3 = \frac{k_3^*}{c_{\text{tot}}}, \quad k_c = \frac{k_c^*}{k_5^*} s_{\text{max}} c_{\text{tot}}, \quad k_4 = \frac{k_4^*}{k_5^* b_{\text{tot}}}, \quad \tau_b = \tau_b^* k_5^*,$$

and (2) becomes

$$\frac{dc}{dt} = k_1 b(1-c) - k_2 c + k_3, \tag{3}$$
$$\frac{db}{dt} = (k_c sc(1-b) - b + k_4)\tau_b.$$

Conditions for a "switch-like" response When the stimulus s goes from an "off" state to an "on" state, C usually responds in a similar fashion. In order for the two response states in C to separate significantly like a "switch", the following three conditions must be satisfied:

- $k_4 \ll 1$, and $k_3 \ll k_2$. This set of constraints means the basal ativation levels of C and B must be relatively low compared to the deactivation rate of C and B, respectively.
- $k_1/k_2 \ll 1/k_4$. This implies that when the signal is off, the activation from B to C, which is a product of k_1 and k_4 (the level of B at $s = 0$) must be significantly less than the deactivation of C, k_2.
- $k_1/k_2 > 1/k_c$. This suggests that the strength of activation from B to C, measured by k_1/k_2, is greater than the deactivation of B to produce C, measured by $1/k_c$. That is, more active C should be produced from B than the C that participates in the activation of B.

The second and third conditions together imply $k_4 \ll k_c$, that is, B should be mainly activated through

C not from basal activation. The details on derivation of the three conditions are as follows. We use $(\bar{\cdot})$ to denote the steady steady value of a variable. At the steady state, the right hand side of system (3) equals zero, yielding

$$\bar{b} = \frac{k_c s\bar{c} + k_4}{k_c s\bar{c} + 1},$$

where $\bar{c}$ satisfies

$$A_2\bar{c}^2 + A_1\bar{c} - A_0 = 0, \tag{4}$$

with

$$A_2 = (k_1 + k_2)k_c s, \quad A_1 = k_2 - k_3k_c s - k_1k_c s + k_1k_4, \quad A_0 = k_1k_4 + k_3.$$

Let $\bar{c}_0$ and $\bar{b}_0$ ($\bar{c}_1$ and $\bar{b}_1$) denote the steady states of c and b at $s = 0$ ($s = 1$), respectively. When $s = 0$, equation (4) is linear, and thus

$$\bar{c}_0 = \frac{k_1k_4 + k_3}{k_1k_4 + k_2}, \quad \bar{b}_0 = k_4.$$

It is clear that $\bar{c}_0$ and $\bar{b}_0$ are close to zero provided

$$k_4 \ll 1, \quad k_3 \ll k_2, \quad k_1k_4 \ll k_2. \tag{5}$$

When $s \neq 0$, (4) is a quadratic equation with two real roots of different signs. The positive root can be written as

$$\bar{c} = \frac{-A_1 + \sqrt{A_1^2 + 4A_2A_0}}{2A_2}. \tag{6}$$

Under condition (5) and the assumption $k_1 \gg k_3$, we have $A_1 \gg A_0$ and that A_1 and A_2 are about the same order (they both contain terms like k_2 and $k_1k_c s$). Thus, $A_1^2 + 4A_2A_0 \approx A_1^2$, and depending on the sign of A_1 there are two cases of $\bar{c}$ at $s = 1$.

1. $k_1k_c \leq k_2$. In this case, $\sqrt{A_1^2 + 4A_2A_0} \approx A_1$, and by (6), $\bar{c}_1 \approx 0$, which is close to the inactive steady state $\bar{c}_0$, violating our "switch-like" response assumption. This unrealistic case should be avoided.

2. $k_1 k_c > k_2$. In this case, $\sqrt{A_1^2 + 4A_2 A_0} \approx -A_1$, and by (6),

$$\bar{c}_1 \approx \frac{-2A_1}{2A_2} \approx \frac{k_1 k_c - k_2}{(k_1 + k_2)k_c}. \tag{7}$$

We remark that the same condition is also obtained from the Fluctuation Dissipation Theorem approach in Section 5. The corresponding steady state value of b at $s = 1$ is

$$\bar{b}_1 \approx \frac{k_1 k_c - k_2}{k_1 k_c + k_1}. \tag{8}$$

Let us denote J as the Jacobian matrix of system (3):

$$J(s, c, b) = \begin{pmatrix} -k_1 b - k_2 & k_1(1-c) \\ k_c s(1-b)\tau_b & -(k_c s c + 1)\tau_b \end{pmatrix}.$$

1.1 The deactivation time scale

Our goal here is to estimate the time scale $t_{1\to 0}$ and to find the parameters that controls $t_{1\to 0}$. We start by defining the deactivation process. Suppose that system (3) is well stabilized at the active state, that is, the initial condition of system (3) is $(\bar{c}_1, \bar{b}_1)$, and then we apply the signal $s(t) = 0, t \geq 0$. The process from $t = 0$ till the output is stabilized around the equilibrium $\bar{c}_0$ is called the deactivation process. The deactivation time scale $t_{1\to 0}$ is defined as the time when $c(t)$ reaches $(\bar{c}_1 - \bar{c}_0)/e + \bar{c}_0$. During the whole deactivation process, system (3) sees only signal $s \equiv 0$, so we linearize (3) around the steady state at $s = 0$:

$$\begin{pmatrix} \delta c(t) \\ \delta b(t) \end{pmatrix}' = J(0, \bar{c}_0, \bar{b}_0) \begin{pmatrix} \delta c(t) \\ \delta b(t) \end{pmatrix}, \tag{9}$$

where

$$J(0, \bar{c}_0, \bar{b}_0) = \begin{pmatrix} -k_1 \bar{b}_0 - k_2 & k_1(1 - \bar{c}_0) \\ 0 & -\tau_b \end{pmatrix}.$$

Clearly, the eigenvalues of $J(0, \bar{c}_0, \bar{b}_0)$ are

$$\lambda_c = -(k_1 k_4 + k_2), \quad \lambda_b = -\tau_b,$$

and the corresponding eigenvectors are

$$\xi_c = \begin{pmatrix} 1 \\ 0 \end{pmatrix}, \quad \xi_b = \begin{pmatrix} \frac{k_1(1-\bar{c}_0)}{k_1 k_4 + k_2 - \tau_b} \\ 1 \end{pmatrix}.$$

Solutions of system (9) can be written as linear combination of $\xi_c e^{-k_2 t}, \quad \xi_b e^{-\tau_b t}$, that is,

$$\delta c(t) = l_c \xi_c^1 e^{-(k_1 k_4 + k_2)t} + l_b \xi_b^1 e^{-\tau_b t}, \tag{10}$$

$$\delta b(t) = l_b \xi_b^2 e^{-\tau_b t}, \tag{11}$$

for some constants l_c and l_b depending on initial conditions [11]. Here, ξ_b^1 denotes the first coordinate of ξ_b.

From (10)-(11), it is easy to see that without loop B the time scale of C is around $1/k_2$, whereas with loop B the time scale would be determined by the larger one between $1/k_2$ and $1/\tau_b$. Therefore, in order to obtain a much slower time scale of C when B is present, τ_b needs to be much smaller than k_2. From now on, we assume that $\tau_b \ll k_2$. Note that when $\tau_b \ll k_2$,

$$\xi_b^1 \approx \frac{k_1(k_2 - k_3)}{(k_1 k_4 + k_2)^2}.$$

For an arbitrary initial condition (γ_0^*, β_0^*), we have

$$l_c \approx \gamma_0^* - \beta_0^* \frac{k_1(k_2 - k_3)}{(k_1 k_4 + k_2)^2}, \quad l_b = \beta_0^*,$$

and thus

$$\delta c(t) \approx \left(\gamma_0^* - \beta_0^* \frac{k_1(k_2-k_3)}{(k_1k_4+k_2)^2}\right) e^{-(k_1k_4+k_2)t} + \beta_0^* \frac{k_1(k_2-k_3)}{(k_1k_4+k_2)^2} e^{-\tau_b t}. \tag{12}$$

On the other hand, under the assumption $\tau_b \ll k_2$, there is a time scale separation in (10). Throughout the initial layer (the time interval on the order of $1/k_2$), we can view $l_b\xi_b^1 e^{-\tau_b t}$ as a constant $l_b\xi_b^1$, while the first term in (10) decays quickly. As a result, after the initial layer, δc falls from its initial condition c_0 to a neighborhood of $l_b\xi_b^1$. If $l_b\xi_b^1$ is small comparing to c_0, then δc has changed substantially during the initial layer, and thus the effect from $e^{-\tau_b t}$ is diminished. Consider the extreme case when $l_b\xi_b^1$ is below $(\bar{c}_1 - \bar{c}_0)/e$. The deactivation time scale $t_{1\to 0}$ is defined as the time when $c(t)$ reaches $(\bar{c}_1 - \bar{c}_0)/e + \bar{c}_0$, which when linearized around $\bar{c}_0$ corresponds to $\delta c(t)$ being $(\bar{c}_1 - \bar{c}_0)/e$. If $l_b\xi_b^1$ is below $(\bar{c}_1 - \bar{c}_0)/e$, then δc reaches $(\bar{c}_1 - \bar{c}_0)/e$ during the initial layer, i.e., $t_{1\to 0}$ is within the order of $1/k_2$. So when τ_b is small, the deactivation process may not be significantly affected by the term $e^{-\tau_b t}$. Conversely, if $l_b\xi_b^1/\gamma_0^*$ is large, $e^{-\tau_b t}$ will have substantial contribution in determining $t_{1\to 0}$. We remark that it is possible in general (for example, the positive-negative-loop case) to have $l_b\xi_b^1$ greater than the initial state (the active state), and in that case $\delta c(t)$ increases during the initial layer, resulting in a even longer period for $\delta c(t)$ to reach $(\bar{c}_1 - \bar{c}_0)/e$. In order to estimate $l_b\xi_b^1$, notice that $l_b\xi_b^2 = \beta_0^*$ and $l_b\xi_b^1/\gamma_0^* = (\beta_0^*\xi_b^1)/(\gamma_0^*\xi_b^2)$. Therefore, for any fixed initial condition (γ_0^*, β_0^*) around the steady state $(0,0)$, the ratio ξ_b^1/ξ_b^2 determines how effectively $e^{-\tau_b t}$ slows down the dynamics of δc. Calculating this ratio, we obtain

$$\frac{\xi_b^1}{\xi_b^2} = \frac{k_1(1-\bar{c}_0)}{k_1k_4+k_2-\tau_b} \overset{\tau_b \ll k_2}{\approx} \frac{k_1(k_2-k_3)}{(k_1k_4+k_2)^2} \overset{k_3 \ll k_2}{\approx} \frac{k_1k_2}{(k_1k_4+k_2)^2} \overset{k_1k_4 \ll k_2}{\approx} \frac{k_1k_2}{k_2^2} = K_a.$$

The bigger K_a is, the more contribution from the slow term $e^{-\tau_b t}$, and thus the slower δc converges to the steady state at zero.

To estimate $t_{1\to 0}$, we consider the initial condition $(\gamma_0^*, \beta_0^*) = (\bar{c}_1 - \bar{c}_0, \bar{b}_1 - \bar{b}_0)$, and compute the time when δc reaches γ_0^*/e. If $l_b\xi_b^1/\gamma_0^*$ is large, the initial layer can be neglected, and $\delta c(t) \approx l_b\xi_b^1 e^{-\tau_b t}$. The time

it takes for δc to drop from γ_0^* to γ_0^*/e is approximately,

$$\frac{1+\ln\left(\frac{l_b\xi_b^1}{\gamma_0^*}\right)}{\tau_b} = \frac{1+\ln\left(\frac{\xi_b^1\beta_0^*}{\xi_b^2\gamma_0^*}\right)}{\tau_b}.$$

So, the quantity, $\frac{\xi_b^1\beta_0^*}{\xi_b^2\gamma_0^*}$ affects the duration of the deactivation process, with bigger $\frac{\xi_b^1\beta_0^*}{\xi_b^2\gamma_0^*}$ corresponding to longer deactivation. Notice that,

$$\frac{\beta_0^*}{\gamma_0^*} = \frac{\bar{b}_1-\bar{b}_0}{\bar{c}_1-\bar{c}_0} \approx \left(\frac{K_a+1}{Ka}\right)\left(\frac{k_c}{k_c+1}\right).$$

Thus,

$$\frac{\xi_b^1\beta_0^*}{\xi_b^2\gamma_0^*} \approx (K_a+1)\frac{k_c}{k_c+1}. \tag{13}$$

Therefore, $t_{1\to 0}$ is increasing in K_a and k_c. The above linear approximation (10)-(11) is only valid around a small neighborhood of $(0,0)$, and the global dynamics could be different. However, demonstrated by numerical simulations, the linear analysis seems to provide good qualitative predictions of the dynamics and the dependence on K_a and k_c (Figures 3E-3F). For the sake of completeness, we also used the two-time-scale asymptotic expansion of the global solution (Section 4) and the Fluctuation Dissipation Theorem (Section 5). Both approaches reinforced the conclusions obtained by the linear analysis.

1.2 The activation time scale

Suppose that system (3) is well stabilized at the inactive state, at time zero, apply the signal $s(t)=1, t\geq 0$. The process from $t=0$ till the system is stabilized around the equilibrium $\bar{c}_1$ is defined as the activation process. During activation, the input signal is $s\equiv 1$, so we linearize (3) around the steady state at $s=1$:

$$\begin{pmatrix}\delta c(t)\\ \delta b(t)\end{pmatrix}' = J(1,\bar{c}_1,\bar{b}_1)\begin{pmatrix}\delta c(t)\\ \delta b(t)\end{pmatrix},$$

where

$$J(1, \bar{c}_1, \bar{b}_1) = \begin{pmatrix} -k_1\bar{b}_1 - k_2 & k_1(1-\bar{c}_1) \\ k_c(1-\bar{b}_1)\tau_b & -(k_c\bar{c}_1+1)\tau_b \end{pmatrix}.$$

Let us assume that the term $k_c(1-\bar{b}_1)\tau_b$ is small enough ($\tau_b \ll k_2$) so that the eigenvalues of $J(1, \bar{c}_1, \bar{b}_1)$ are

$$\lambda_c \approx -(k_1\bar{b}_1 + k_2), \quad \lambda_b \approx -(k_c\bar{c}_1 + 1)\tau_b,$$

and their corresponding eigenvectors are

$$\xi_c \approx \begin{pmatrix} 1 \\ 0 \end{pmatrix}, \quad \xi_b \approx \begin{pmatrix} \frac{k_1(1-\bar{c}_1)}{k_1\bar{b}_1+k_2-(k_c\bar{c}_1+1)\tau_b} \\ 1 \end{pmatrix}.$$

Therefore,

$$\begin{aligned} \delta c(t) &\approx l_c\xi_c^1 e^{-(k_1\bar{b}_1+k_2)t} + l_b\xi_b^1 e^{-(k_c\bar{c}_1+1)\tau_b t}, \\ \delta b(t) &\approx l_b\xi_b^2 e^{-(k_c\bar{c}_1+1)\tau_b t}, \end{aligned} \tag{14}$$

for some constants l_c and l_b depending on initial conditions. If $(k_c\bar{c}_1 + 1)\tau_b \ll k_2$, there is a time scale separation in the solution of δc. Similar to the analysis for the deactivation time scale, if the ratio

$$\frac{\xi_b^1}{\xi_b^2} \approx \frac{k_1(1-\bar{c}_1)}{k_1\bar{b}_1 + k_2} = \frac{K_a}{(K_a+1)^2}\left(1 + \frac{1}{k_c}\right)^2,$$

is large, then $e^{-(k_c\bar{c}_1+1)\tau_b t}$ determines the time scale of the convergence to zero. To estimate $t_{0\to1}$, we consider the initial condition $(\gamma_0^*, \beta_0^*) = (\bar{c}_0 - \bar{c}_1, \bar{b}_0 - \bar{b}_1)$, and define $t_{0\to1}$ as the time when δc reaches γ_0^*/e. If $l_b\xi_b^1/\gamma_0^*$ is large, then the initial layer can be neglected, and $\delta c(t) \approx l_b\xi_b^1 e^{-(k_c\bar{c}_1+1)\tau_b t}$. The time it takes for δc to drop from γ_0^* to γ_0^*/e is

$$\frac{1 + \ln\left(\frac{\xi_b^1\beta_0^*}{\xi_b^2\gamma_0^*}\right)}{\tau_b},$$

where

$$\frac{\xi_b^1 \beta_0^*}{\xi_b^2 \gamma_0^*} \approx \frac{1}{K_a + 1} \left(1 + \frac{1}{k_c} \right). \tag{15}$$

So, $t_{0 \to 1}$ is decreasing in K_a and k_c.

2 Positive-Positive-Loop Module

Recall the positive-positive-loop model:

$$\begin{aligned}
\frac{dc}{dt} &= k_1(a+b)(1-c) - k_2 c + k_3, \\
\frac{da}{dt} &= (k_c sc(1-a) - a + k_4)\tau_a, \\
\frac{db}{dt} &= (k_c sc(1-b) - b + k_4)\tau_b.
\end{aligned} \tag{16}$$

We have similar conditions for a "switch-like" response as in the single-positive-loop case:

- $k_4 \ll 1$, and $k_3 \ll k_2$. This set of constraints means the basal activation levels of C and B must be relatively low compared to the deactivation rate of C and B, respectively.
- $2k_1/k_2 \ll 1/k_4$. This implies that when the signal is off, the activation from A and B to C, which is a product of $2k_1$ and k_4, the level of A or B at $s = 0$, must be significantly less than the deactivation of C, k_2.
- $2k_1/k_2 > 1/k_c$. This suggests that the strength of activation from A and B to C, measured by $2k_1/k_2$, is greater than the deactivation of B to produce active C, measured by $1/k_c$. That is, more C should be activated from A and B than the C that participates in the activation of A and B.

The second and the third conditions together imply $k_4 \ll k_c$, that is, A and B should be mainly activated through C not from basal activation. The details on derivation of the three conditions are as follows. At

the steady state of system (16), we have

$$\bar{a} = \bar{b} = \frac{k_c s \bar{c} + k_4}{k_c s \bar{c} + 1},$$

where $\bar{c}$ satisfies

$$A_2 \bar{c}^2 + A_1 \bar{c} - A_0 = 0, \tag{17}$$

with

$$A_2 = (2k_1 + k_2) k_c s, \quad A_1 = k_2 - k_3 k_c s - 2k_1 k_c s + 2k_1 k_4, \quad A_0 = 2k_1 k_4 + k_3.$$

When $s = 0$, equation (17) is linear, and the steady state is

$$\bar{c}_0 = \frac{2k_1 k_4 + k_3}{2k_1 k_4 + k_2}, \quad \bar{a}_0 = k_4, \quad \bar{b}_0 = k_4.$$

The values of $\bar{c}_0, \bar{a}_0$, and $\bar{b}_0$ are close to zero provided

$$k_4 \ll 1, \quad k_3 \ll k_2, \quad 2k_1 k_4 \ll k_2. \tag{18}$$

When $s \neq 0$, (17) is a quadratic equation with two real roots of different signs. The positive root can be written as

$$\bar{c} = \frac{-A_1 + \sqrt{A_1^2 + 4A_2 A_0}}{2A_2}. \tag{19}$$

Similar to the single-positive-loop case, under condition (18), there are two cases at $s = 1$.

1. $2k_1 k_c \leq k_2$. In this case, the positive root $\bar{c}_1$ as in (19) is close to zero, and thus can not be differentiated from $\bar{c}_0$. We discard this unrealistic case.

2. $2k_1 k_c > k_2$. In this case, $\sqrt{A_1^2 + 4A_2 A_0} \approx -A_1$, and thus,

$$\bar{c}_1 \approx \frac{2k_1 k_c - k_2}{(2k_1 + k_2) k_c}.$$

The corresponding steady state values of a and b are given by

$$\bar{a}_1 = \bar{b}_1 \approx \frac{2k_1k_c - k_2}{2k_1(k_c+1)}.$$

The Jacobian matrix of system (16) is

$$J(s,c,a,b) = \begin{pmatrix} -k_1(a+b)-k_2 & k_1(1-c) & k_1(1-c) \\ k_cs(1-a)\tau_a & -(k_csc+1)\tau_a & 0 \\ k_cs(1-b)\tau_b & 0 & -(k_csc+1)\tau_b \end{pmatrix}.$$

2.1 The deactivation time scale

Similar to the single-positive-loop analysis, we linearize system (16) around the steady state $(\bar{c}_0, \bar{a}_0, \bar{b}_0)$ and obtain the following system:

$$\begin{pmatrix} \delta c(t) \\ \delta a(t) \\ \delta b(t) \end{pmatrix}' = J(0, \bar{c}_0, \bar{a}_0, \bar{b}_0) \begin{pmatrix} \delta c(t) \\ \delta a(t) \\ \delta b(t) \end{pmatrix}. \tag{20}$$

The eigenvalues of $J(0, \bar{c}_0, \bar{a}_0, \bar{b}_0)$ are

$$\lambda_c = -(2k_1k_4 + k_2) \approx -k_2, \quad \lambda_a = -\tau_a, \quad \lambda_b = -\tau_b.$$

The corresponding eigenvectors are

$$\xi_c = \begin{pmatrix} 1 \\ 0 \\ 0 \end{pmatrix}, \quad \xi_a = \begin{pmatrix} \frac{k_1(1-\bar{c}_0)}{2k_1k_4+k_2-\tau_a} \\ 1 \\ 0 \end{pmatrix}, \quad \xi_b = \begin{pmatrix} \frac{k_1(1-\bar{c}_0)}{2k_1k_4+k_2-\tau_b} \\ 0 \\ 1 \end{pmatrix}.$$

Solutions of system (20) can be written as linear combinations of

$$\xi_c e^{-k_2 t}, \ \ \xi_a e^{-\tau_a t}, \ \ \xi_b e^{-\tau_b t},$$

that is,

$$\begin{aligned} \delta c(t) &= l_c \xi_c^1 e^{-k_2 t} + l_a \xi_a^1 e^{-\tau_a t} + l_b \xi_b^1 e^{-\tau_b t}, \\ \delta a(t) &= l_a \xi_a^2 e^{-\tau_a t}, \\ \delta b(t) &= l_b \xi_b^3 e^{-\tau_b t}, \end{aligned} \tag{21}$$

for some constants l_c, l_a, and l_b depending on initial conditions.

From (21), we see that the largest number among $1/k_2$, $1/\tau_a$, and $1/\tau_b$ indicates the time scale of δc. In order to observe a slow deactivation, one of τ_a and τ_b must be much smaller than k_2. Without loss of generality, we assume that $\tau_b \ll k_2$ and $\tau_b \leq \tau_a$ (τ_a and τ_b are symmetric).

For the same reason as the single-positive-loop system, for any fixed initial condition in a small neighborhood of the stead state, the ratio of ξ_b^1 to ξ_b^3 determines how effectively $e^{-\tau_b t}$ slows down the dynamics of δc. Calculating the ratio, we obtain

$$\frac{\xi_b^1}{\xi_b^3} = \frac{k_1(1-\bar{c}_0)}{2k_1k_4 + k_2 - \tau_b} \overset{\tau_b \ll k_2}{\approx} \frac{k_1(k_2 - k_3)}{(2k_1k_4 + k_2)^2} \overset{k_3 \ll k_2}{\approx} \frac{k_1 k_2}{(2k_1k_4 + k_2)^2} \overset{2k_1k_4 \ll k_2}{\approx} \frac{k_1 k_2}{k_2^2} = K_a.$$

Thus, the bigger K_a is, the slower δc converges to the zero steady state. To estimate $t_{1\to 0}$, we consider the initial condition $(\gamma_0^*, \alpha_0^*, \beta_0^*) = (\bar{c}_1 - \bar{c}_0, \bar{a}_1 - \bar{a}_0, \bar{b}_1 - \bar{b}_0)$, and take $t_{1\to 0}$ as the time when δc reaches γ_0^*/e. We use two extreme cases of τ_a to illustrate the effect of loop A on the deactivation time scale $t_{1\to 0}$.

Case 1 $\tau_a \approx k_2 \gg \tau_b$. After an initial period, $\delta c(t) \approx l_b \xi_b^1 e^{-\tau_b t}$. If $l_b \xi_b^1$ is significant comparing to γ_0^*, then

the time it takes for δc to drop from γ_0^* to γ_0^*/e is approximately

$$\frac{1+\ln\left(\frac{l_b\xi_b^1}{\gamma_0^*}\right)}{\tau_b} = \frac{1+\ln\left(\frac{\xi_b^1\beta_0^*}{\xi_b^3\gamma_0^*}\right)}{\tau_b},$$

where

$$\frac{\xi_b^1}{\xi_b^3} = \frac{k_1(1-\bar{c}_0)}{2k_1k_4+k_2-\tau_b} \overset{\tau_b \ll k_2}{\approx} \frac{k_1(k_2-k_3)}{(2k_1k_4+k_2)^2} \overset{2k_1k_4 \ll k_2}{\approx} \frac{k_1k_2}{k_2^2} = K_a,$$

and

$$\frac{\beta_0^*}{\gamma_0^*} \approx \left(\frac{2K_a+1}{2K_a}\right)\left(\frac{k_c}{k_c+1}\right).$$

Thus,

$$\frac{\xi_b^1\beta_0^*}{\xi_b^3\gamma_0^*} \approx (K_a+\frac{1}{2})\frac{k_c}{1+k_c}. \tag{22}$$

Comparing (22) with (13), we see that the $t_{1\to 0}$ in the positive-positive-loop system is smaller. On the other hand, in both single-positive-loop and positive-positive-loop systems, $t_{1\to 0}$ increases in K_a and k_c.

Case 2 $k_2 \gg \tau_a = \tau_b$. The solutions in (21) can be rewritten as

$$\delta c(t) = l_c\xi_c^1 e^{-k_2 t} + 2l_b\xi_b^1 e^{-\tau_b t},$$

$$\delta b(t) = l_b\xi_b^3 e^{-\tau_b t},$$

$$\delta a(t) = \delta b(t).$$

After the initial layer, $\delta c(t) \approx 2l_b\xi_b^1 e^{-\tau_b t}$. If $2l_b\xi_b^1/\gamma_0^*$ is large, then the time it takes for δc to drop from γ_0^* to γ_0^*/e is

$$\frac{1+\ln\left(\frac{2\xi_b^1\beta_0^*}{\xi_b^3\gamma_0^*}\right)}{\tau_b},$$

where

$$\frac{2\xi_b^1\beta_0^*}{\xi_b^3\gamma_0^*} \approx (2K_a+1)\frac{k_c}{1+k_c}. \tag{23}$$

Comparing (22) to (13), we see that the $t_{1\to 0}$ in the positive-positive-loop system is bigger.

The analysis of the above two extreme cases suggests that with the additional loop A, the deactivation time scale $t_{1\to 0}$ of the positive-positive-loop system can be either smaller or bigger than the single-positive-loop case depending on τ_a in a manner that bigger τ_a corresponding to smaller $t_{1\to 0}$. In addition, $t_{1\to 0}$ is increasing in K_a and k_c.

2.2 The activation time scale

Linearizing system (16) around the steady state $(\bar{c}_1, \bar{a}_1, \bar{b}_1)$, we obtain the following system:

$$\begin{pmatrix} \delta c(t) \\ \delta a(t) \\ \delta b(t) \end{pmatrix}' = J(1, \bar{c}_1, \bar{a}_1, \bar{b}_1) \begin{pmatrix} \delta c(t) \\ \delta a(t) \\ \delta b(t) \end{pmatrix},$$

where

$$J(1, \bar{c}_1, \bar{a}_1, \bar{b}_1) = \begin{pmatrix} -k_1(\bar{a}_1 + \bar{b}_1) - k_2 & k_1(1-\bar{c}_1) & k_1(1-\bar{c}_1) \\ k_c(1-\bar{a}_1)\tau_a & -(k_c\bar{c}_1 + 1)\tau_a & 0 \\ k_c(1-\bar{b}_1)\tau_b & 0 & -(k_c\bar{c}_1+1)\tau_b \end{pmatrix}.$$

Let us assume that the term $k_c(1-\bar{b}_1)\tau_b$ is small enough so that $J(1, \bar{c}_1, \bar{a}_1, \bar{b}_1) \approx J^*$, where

$$J^* = \begin{pmatrix} -k_1(\bar{a}_1 + \bar{b}_1) - k_2 & k_1(1-\bar{c}_1) & k_1(1-\bar{c}_1) \\ k_c(1-\bar{a}_1)\tau_a & -(k_c\bar{c}_1 + 1)\tau_a & 0 \\ 0 & 0 & -(k_c\bar{c}_1+1)\tau_b \end{pmatrix}.$$

Denote the eigenvalues of J^* as

$$\lambda_c, \quad \lambda_a, \quad \lambda_b = -(k_c\bar{c}_1 + 1)\tau_b,$$

with their corresponding eigenvectors

$$\xi_c \approx \begin{pmatrix} \xi_c^1 \\ \xi_c^2 \\ 0 \end{pmatrix}, \quad \xi_a \approx \begin{pmatrix} \xi_a^1 \\ \xi_a^2 \\ 0 \end{pmatrix}, \quad \xi_b \approx \begin{pmatrix} \frac{k_1(1-\bar{c}_1)}{k_1(\bar{a}_1+\bar{b}_1)+k_2-(k_c\bar{c}_1+1)\tau_b} \\ 0 \\ 1 \end{pmatrix}.$$

To estimate $t_{0\to 1}$, we consider the initial condition $(\gamma_0^*, \alpha_0^*, \beta_0^*) = (\bar{c}_0 - \bar{c}_1, \bar{a}_0 - \bar{a}_1, \bar{b}_0 - \bar{b}_1)$. Again, we use the previous two extreme cases of τ_a to elucidate the effect of the loop A on $t_{0\to 1}$.

Case 1 $\tau \approx k_2 \gg \tau_b$. After an initial period, δc can be approximated by $l_b \xi_b^1 e^{-\tau_b t}$. If $l_b \xi_b^1/\gamma_0^*$ is large, then $t_{1\to 0}$ can be approximated by

$$\frac{1 + \ln\left(\frac{\xi_b^1 \beta_0^*}{\xi_b^3 \gamma_0^*}\right)}{\tau_b},$$

where

$$\frac{\xi_b^1}{\xi_b^3} \approx \frac{k_1 k_2}{(2k_1 + k_2)^2}\left(1 + \frac{1}{k_c}\right)^2 = \frac{K_a}{\left(2K_a + 1\right)^2}\left(1 + \frac{1}{k_c}\right)^2, \quad \frac{\beta_0^*}{\gamma_0^*} \approx \left(\frac{2K_a + 1}{2K_a}\right)\left(\frac{k_c}{k_c + 1}\right).$$

Thus,

$$\frac{\xi_b^1 \beta_0^*}{\xi_b^3 \gamma_0^*} \approx \frac{1}{2(2K_a + 1)^2}\left(1 + \frac{1}{k_c}\right). \tag{24}$$

The ratio in (24) is much smaller than that in (15). On the other hand, in both single-positive-loop and positive-positive-loop systems, $t_{0\to 1}$ is decreasing in K_a and k_c.

Case 2 $k_2 \gg \tau_a = \tau_b$. After the initial layer, $\delta c \approx 2 l_b \xi_b^1 e^{-\tau_b t}$. If $2 l_b \xi_b^1/\gamma_0^*$ is significant, then the time it takes for δc to reach γ_0^*/e is

$$\frac{1 + \ln\left(\frac{2\xi_b^1 \beta_0^*}{\xi_b^3 \gamma_0^*}\right)}{\tau_b},$$

where

$$\frac{2\xi_b^1 \beta_0^*}{\xi_b^3 \gamma_0^*} \approx \frac{1}{(2K_a + 1)^2}\left(1 + \frac{1}{k_c}\right). \tag{25}$$

Comparing (25) to (15), we see that the $t_{1\to 0}$ in the positive-positive-loop system is smaller.

The analysis of the above two extreme cases suggests that with the additional loop A, the activation time scale $t_{1\to 0}$ is smaller than that in the single-positive-loop case. Moreover, $t_{0\to 1}$ is decreasing in K_a and k_c.

3 Positive-Negative-Loop Module

Recall the equations for the positive-negative-loop module:

$$\begin{aligned}\frac{dc}{dt} &= k_{1b}b(1-c) - (k_2 + k_{1a}a)c + k_3,\\ \frac{da}{dt} &= (k_{ca}sc(1-a) - a + k_4)\tau_a,\\ \frac{db}{dt} &= (k_{cb}sc(1-b) - b + k_4)\tau_b.\end{aligned} \tag{26}$$

For the purpose of analysis, we assume that $k_{ca} = k_{cb} := k_c$, which significantly simplifies our computations. The steady state at $s = 0$ is

$$\bar{c}_0 = \frac{k_3 + k_{1b}k_4}{k_2 + k_4(k_{1a} + k_{1b})}, \quad \bar{a}_0 = \bar{b}_0 = k_4.$$

When $s = 1$, under the assumptions $k_4 \ll 1$, $k_3 \ll k_2$, and $k_{1b}k_c > k_2$ (similar to those in the positive-positive-loop systems, Section 2), we have

$$\bar{c}_1 \approx \frac{k_{1b}k_c - k_2}{k_2k_c + (k_{1b} + k_{1a})k_c}, \quad \bar{a}_1 = \bar{b}_1 \approx \frac{k_{1b}k_c - k_2}{k_{1b}k_c + k_{1b} + k_{1a}}.$$

The Jacobian matrix of (26) is

$$J(s,c,a,b) = \begin{pmatrix} -k_{1b}b - (k_2 + k_{1a}a) & -k_{1a}c & k_{1b}(1-c) \\ k_{ca}s(1-a)\tau_a & -(k_{ca}sc+1)\tau_a & 0 \\ k_{cb}s(1-b)\tau_b & 0 & -(k_{cb}sc+1)\tau_b \end{pmatrix}.$$

3.1 The deactivation time scale

Linearizing (26) around the steady state $(\bar{c}_0, \bar{a}_0, \bar{b}_0)$, we obtain the following system:

$$\begin{pmatrix} \delta c(t) \\ \delta a(t) \\ \delta b(t) \end{pmatrix}' = J(0, \bar{c}_0, \bar{a}_0, \bar{b}_0) \begin{pmatrix} \delta c(t) \\ \delta a(t) \\ \delta b(t) \end{pmatrix}. \tag{27}$$

The eigenvalues of $J(0, \bar{c}_0, \bar{a}_0, \bar{b}_0)$ are

$$\lambda_c = -((k_{1b} + k_{1a})k_4 + k_2) \approx -k_2, \quad \lambda_a = -\tau_a, \quad \lambda_b = -\tau_b.$$

The corresponding eigenvectors are

$$\xi_c = \begin{pmatrix} 1 \\ 0 \\ 0 \end{pmatrix}, \quad \xi_a = \begin{pmatrix} -\frac{k_{1a}\bar{c}_0}{(k_{1b}+k_{1a})k_4+k_2-\tau_a} \\ 1 \\ 0 \end{pmatrix}, \quad \xi_b = \begin{pmatrix} \frac{k_{1b}(1-\bar{c}_0)}{(k_{1b}+k_{1a})k_4+k_2-\tau_b} \\ 0 \\ 1 \end{pmatrix}.$$

Solutions of system (27) can be written as the linear combination of

$$\xi_c e^{-k_2 t}, \quad \xi_a e^{-\tau_a t}, \quad \xi_b e^{-\tau_b t},$$

that is,

$$\begin{aligned} \delta c(t) &= l_c \xi_c^1 e^{-k_2 t} + l_a \xi_a^1 e^{-\tau_a t} + l_b \xi_b^1 e^{-\tau_b t}, \\ \delta a(t) &= l_a \xi_a^2 e^{-\tau_a t}, \\ \delta b(t) &= l_b \xi_b^3 e^{-\tau_b t}, \end{aligned} \tag{28}$$

for some constants l_c, l_a, and l_b depending on initial conditions. As done for the positive-positive-loop system, we consider the following two cases.

Case 1 $\tau_a \approx k_2 \gg \tau_b$, fast negative loop and slow positive loop. The contribution of the slow dynamics is through the term $l_b \xi_b^1 e^{-\tau_b t}$. As before, we compute

$$\frac{\xi_b^1}{\xi_b^3} = \frac{k_{1b}(1-\bar{c}_0)}{(k_{1b}+k_{1a})k_4 + k_2 - \tau_b} \overset{\tau_b \ll k_2}{\approx} \frac{k_{1b}(k_2 + k_{1a}k_4 - k_3)}{((k_{1b}+k_{1a})k_4 + k_2)^2} \overset{k_3,k_4 \ll k_2}{\approx} \frac{k_{1b}}{k_2} := K_a,$$

and

$$\frac{\beta_0^*}{\gamma_0^*} \approx \frac{(1+K_a+K_d)k_c}{K_a + K_a k_c + K_d}.$$

Thus,

$$\frac{\xi_b^1 \beta_0^*}{\xi_b^3 \gamma_0^*} \approx \frac{(1+K_a+K_d)k_c}{k_c + 1 + K_d/K_a}. \tag{29}$$

A straightforward calculation shows that (29) is always greater than (13) under the assumption $k_{1b}k_c > k_2$ (the same assumption that guarantees the positivity of $\bar{c}_1$). Equation (29) also shows that $t_{1\to 0}$ increases in K_a and k_c.

Case 2 $k_2 \gg \tau_a = \tau_b$, slow negative loop and slow positive loop. In this case, both terms $l_a \xi_a^1 e^{-\tau_a t}$ and $l_b \xi_b^1 e^{-\tau_b t}$ are slow. The overall contribution from the slow terms is indicated by $\frac{(\xi_b^1+\xi_a^1)\beta_0^*}{\xi_b^2\gamma_0^*}$. Thus,

$$\frac{(\xi_b^1+\xi_a^1)\beta_0^*}{\xi_b^2\gamma_0^*} \approx \frac{(1+K_a+K_d)k_c}{k_c+1+K_d/K_a} - K_d\left(\frac{k_3}{k_2} + K_a k_4\right)\frac{(1+K_a+K_d)k_c}{K_a+K_a k_c+K_d}. \tag{30}$$

Notice that $k_4 \ll 1$ and $k_3 \ll k_2$, so the second term in (30) is very small. Thus, (30) is still bigger than (13), but smaller than (29). That is, the deactivation in this case is slower than that in the single-positive-loop case, but faster than the fast-negative-slow-positive case.

3.2 The activation time scale

Linearizing system (26) around the steady state $(\bar{c}_1, \bar{a}_1, \bar{b}_1)$, we obtain the following system:

$$\begin{pmatrix} \delta c(t) \\ \delta a(t) \\ \delta b(t) \end{pmatrix}' = J(1, \bar{c}_1, \bar{a}_1, \bar{b}_1) \begin{pmatrix} \delta c(t) \\ \delta a(t) \\ \delta b(t) \end{pmatrix},$$

where

$$J(1, c, a, b) = \begin{pmatrix} -k_{1b}\bar{b}_1 - (k_2 + k_{1a}\bar{a}_1) & -k_{1a}\bar{c}_1 & k_{1b}(1 - \bar{c}_1) \\ k_{ca}(1 - \bar{a}_1)\tau_a & -(k_{ca}\bar{c}_1 + 1)\tau_a & 0 \\ k_{cb}(1 - \bar{b}_1)\tau_b & 0 & -(k_{cb}\bar{c}_1 + 1)\tau_b \end{pmatrix}.$$

Let us assume that the term $k_{cb}(1 - \bar{b}_1)\tau_b$ is small enough so that $J(1, \bar{c}_1, \bar{a}_1, \bar{b}_1) \approx J^*$, where

$$J^* = \begin{pmatrix} -k_{1b}\bar{b}_1 - (k_2 + k_{1a}\bar{a}_1) & -k_{1a}\bar{c}_1 & k_{1b}(1 - \bar{c}_1) \\ k_{ca}(1 - \bar{a}_1)\tau_a & -(k_{ca}\bar{c}_1 + 1)\tau_a & 0 \\ 0 & 0 & -(k_{cb}\bar{c}_1 + 1)\tau_b \end{pmatrix}.$$

Denote the eigenvalues of J^* as

$$\lambda_c, \quad \lambda_a, \quad \lambda_b = -(k_{cb}\bar{c}_1 + 1)\tau_b,$$

with their corresponding eigenvectors

$$\xi_c \approx \begin{pmatrix} \xi_c^1 \\ \xi_c^2 \\ 0 \end{pmatrix}, \quad \xi_a \approx \begin{pmatrix} \xi_a^1 \\ \xi_a^2 \\ 0 \end{pmatrix}, \quad \xi_b \approx \begin{pmatrix} \frac{k_{1b}(1-\bar{c}_1)}{k_{1b}\bar{b}_1 + (k_2 + k_{1a}\bar{a}_1) - (k_{cb}\bar{c}_1 + 1)\tau_b} \\ 0 \\ 1 \end{pmatrix}.$$

To estimate $t_{0\to1}$, we consider the initial condition $(\gamma_0^*, \alpha_0^*, \beta_0^*) = (\bar{c}_0 - \bar{c}_1, \bar{a}_0 - \bar{a}_1, \bar{b}_0 - \bar{b}_1)$. Again, we use the previous two extreme cases of τ_a to elucidate the effect of the loop A on $t_{0\to1}$.

Case 1 $\tau_a \approx k_2 \gg \tau_b$, fast negative loop and slow positive loop. The slow term is $l_b \xi_b^1 e^{-\tau_b t}$. After an initial period, δc can be approximated by $l_b \xi_b^1 e^{-\tau_b t}$. Calculating ξ_b^1/ξ_b^3, we have

$$\frac{\xi_b^1}{\xi_b^3} \approx \frac{(k_c + k_c K_d + 1)(K_a + K_a k_c + K_d)}{k_c^2 (K_a + K_d + 1)^2},$$

which is consistent with of the single-positive-loop case (corresponding to $K_d = 0$). On the other hand,

$$\frac{\beta_0^*}{\gamma_0^*} \approx \frac{(1 + K_a + K_d) k_c}{K_a + K_a k_c + K_d}.$$

Thus

$$\frac{\xi_b^1 \beta_0^*}{\xi_b^3 \gamma_0^*} \approx \frac{k_c + k_c K_d + 1}{k_c (K_a + K_d + 1)}. \tag{31}$$

It is easy to see that (31) is greater than (15) under the condition $k_{1b} k_c > k_2$. So, in this case we expect slower activation in positive-negative-loop systems than in single-positive-loop systems.

Case 2 $k_2 \gg \tau_a = \tau_b$, slow negative loop and slow positive loop. Now both terms $l_a \xi_a^1 e^{-\tau_a t}$ and $l_b \xi_b^1 e^{-\tau_b t}$ are slow, and the contribution from the slow terms is indicated by

$$\frac{(\xi_b^1 + \xi_a^1)\beta_0^*}{\xi_b^2 \gamma_0^*} \approx \frac{k_c + k_c K_d + 1}{k_c (K_a + K_d + 1)} - \frac{K_d (K_a k_c - 1)}{K_a k_c (K_a + K_d + 1)},$$

which is less than (15). That is, the activation in this case is faster than that in the single-positive-loop system. Simulation shows that the are very close (Figures 5C-5D).

The analysis of the above two extreme cases suggests that with the additional negative loop A, the activation time scale in the positive-negative-loop system could be either slower or faster than that in the single-positive-loop system.

4 Two-Time-Scale Asymptotic Expansion

In this section, we derive an asymptotic expansion of the overall solution, including those far away from the steady state. We will show that the single-positive-loop system can function as a low-pass filter, which explains why the relative size of the noise's time scale and the system's intrinsic time scales is important to noise attenuation. We also show that our local solution closely resembles the global solution.

Different from the traditional expansion approach, the two-time-scale asymptotic expansion method [7], originated from the strained-corrdinate method [7], is mainly used to avoid the accumulative effect and to obtain a solution uniformly valid over a long time interval. In our case, it also provides an explicit relation between the solutions and the two separated time scales, suggesting that the single-positive-loop system can function as a low-pass filter.

4.1 Low-pass filter

This section is devoted to obtain the asymptotic expansion of solutions of system (3) and to show that the single-positive-loop module can function as a low-pass filter. Let $\varepsilon = \tau_b$. If $\varepsilon \ll k_2$, we could expand the solutions of system (3) in powers of ε:

$$c = c_0(t^+, \tilde{t}) + \varepsilon c_1(t^+, \tilde{t}) + \varepsilon^2 c_2(t^+, \tilde{t}) + \cdots ,$$
$$b = b_0(t^+, \tilde{t}) + \varepsilon b_1(t^+, \tilde{t}) + \varepsilon^2 b_2(t^+, \tilde{t}) + \cdots ,$$

involving the fast time scale $t^+ = t$ and the slow time scale $\tilde{t} = \varepsilon t$. The initial conditions are

$$c_0(0,0) = \gamma_0, \quad b_0(0,0) = \beta_0, \quad c_i(0,0) = b_i(0,0) = 0, \quad i = 1, 2, \ldots .$$

Accordingly, system (3) becomes

$$
\begin{aligned}
&\frac{\partial c_0}{\partial t^+} + \varepsilon\left(\frac{\partial c_0}{\partial \tilde{t}} + \frac{\partial c_1}{\partial t^+}\right) + O(\varepsilon^2) \qquad (32)\\
&= k_1\big(b_0(t^+,\tilde{t}) + \varepsilon b_1(t^+,\tilde{t}) + \cdots\big)\big(1 - c_0(t^+,\tilde{t}) - \varepsilon c_1(t^+,\tilde{t}) - \cdots\big) - k_2\big(c_0(t^+,\tilde{t}) + \varepsilon c_1(t^+,\tilde{t}) + \cdots\big) + k_3,\\
&\frac{\partial b_0}{\partial t^+} + \varepsilon\left(\frac{\partial b_0}{\partial \tilde{t}} + \frac{\partial b_1}{\partial t^+}\right) + O(\varepsilon^2) \qquad (33)\\
&= \varepsilon\Big(k_c s(t^+,\tilde{t})\big(c_0(t^+,\tilde{t}) + \varepsilon c_1(t^+,\tilde{t}) + \cdots\big)\big(1 - b_0(t^+,\tilde{t}) - \varepsilon b_1(t^+,\tilde{t}) - \cdots\big)\\
&- \big(b_0(t^+,\tilde{t}) + \varepsilon b_1(t^+,\tilde{t}) + \cdots\big) + k_4\Big).
\end{aligned}
$$

Equating the same order on both sides of each equation, we have

$$\frac{\partial b_0}{\partial t^+} = 0, \text{ i.e. } b_0(t^+,\tilde{t}) = b_0(\tilde{t}), \qquad (34)$$

$$\frac{db_0}{d\tilde{t}} + \frac{\partial b_1}{\partial t^+} = k_c s c_0(1 - b_0) - b_0 + k_4, \qquad (35)$$

and

$$\frac{\partial c_0}{\partial t^+} = k_1 b_0(\tilde{t})(1 - c_0(t^+,\tilde{t})) - k_2 c_0(t^+,\tilde{t}) + k_3, \qquad (36)$$

$$\frac{\partial c_0}{\partial \tilde{t}} + \frac{\partial c_1}{\partial t^+} = k_1(b_1(1 - c_0) - b_0 c_1) - k_2 c_1. \qquad (37)$$

In principal, there are different ways of choosing $b_0(\tilde{t})$. However, in order to obtain a uniform zero-order approximation, we need to choose b_0 such that the solution b_1 from (35) has no linear term in t^+ [7]. Once b_0 is determined, solving c_0 from (36), we obtain

$$
\begin{aligned}
c_0(t^+,\tilde{t}) &= e^{-(k_1 b_0(\tilde{t}) + k_2)t^+}\left(h_0(\tilde{t}) + \frac{k_1 b_0(\tilde{t}) + k_3}{k_1 b_0(\tilde{t}) + k_2}\left(e^{(k_1 b_0(\tilde{t}) + k_2)t^+} - 1\right)\right) \qquad (38)\\
&= \left(\gamma_0 - \frac{k_1 b_0(\tilde{t}) + k_3}{k_1 b_0(\tilde{t}) + k_2}\right) e^{-(k_1 b_0(\tilde{t}) + k_2)t^+} + \frac{k_1 b_0(\tilde{t}) + k_3}{k_1 b_0(\tilde{t}) + k_2}.
\end{aligned}
$$

Thus, the question becomes how to choose an appropriate b_0 for a given noise input. Consider the following scenarios of the input signal:

1. Noise is on a fast time scale.

   In this case, the signal can be written as the sum of a constant input and fast noise term $s_1(t^+)$ with mean zero, i.e. $s = s_0 + s_1(t^+)$. In order to eliminate the linear term of t^+ in the solution of b_1, we choose b_0 satisfying:

$$\frac{db_0}{d\tilde{t}} = k_c s_0(1 - b_0)\frac{k_1 b_0 + k_3}{k_1 b_0 + k_2} - b_0 + k_4. \tag{39}$$

   As a result, (35) becomes

$$\frac{\partial b_1}{\partial t^+} = k_c \left((s_1(t^+) + s_0)c_0 - s_0 \frac{k_1 b_0 + k_3}{k_1 b_0 + k_2} \right) (1 - b_0),$$

   whose the solution b_1 does not contain any linear term of t^+. Notice that (39) is separable and can be solved analytically. Since none of b_1, c_0, and $\partial c_0/\partial \tilde{t}$ contains linear term in t^+ (see (38)), the solution c_1 of (37),

$$c_1(t^+, \tilde{t}) = e^{-(k_1 b_0(\tilde{t}) + k_2)t^+} \int_0^{t^+} \left(k_1 b_1 (1 - c_0) - \frac{\partial c_0}{\partial \tilde{t}} \right) e^{(k_1 b_0(\tilde{t}) + k_2)t^+} dt^+,$$

   has no linear term in t^+. Therefore,

$$\begin{aligned} c(t^+, \tilde{t}) &= \left(\gamma_0 - \frac{k_1 b_0(\tilde{t}) + k_3}{k_1 b_0(\tilde{t}) + k_2} \right) e^{-(k_1 b_0(\tilde{t}) + k_2)t^+} + \frac{k_1 b_0(\tilde{t}) + k_3}{k_1 b_0(\tilde{t}) + k_2} + O(\varepsilon), \\ b(t^+, \tilde{t}) &= b_0(\tilde{t}) + O(\varepsilon), \end{aligned} \tag{40}$$

   is a uniform zero-order approximation of (3) [7] (Figures S3A-S3B). Observe that the noise term $s_1(t^+)$ does not show up in equation (39), so the zero-order approximations with and without noise are the same, suggesting that fast varying noises are filtered out through the system (Figures S3D-S3E).

2. Noise is on the slow time scale $\tilde{t}$, i.e. $s = s(\tilde{t})$.

   In this case, to eliminate the linear term of t^+ in b_1, we take

$$\frac{db_0}{d\tilde{t}} = k_c s(\tilde{t})(1 - b_0)\frac{k_1 b_0 + k_3}{k_1 b_0 + k_2} - b_0 + k_4. \tag{41}$$

   Similar to case 1, we have (40) as the uniform zero-order approximation of (3), but the $b_0(\tilde{t})$ in this case is the solution to (41) instead of (39). Notice that noises persist in the zero-order approximation through the term $s(\tilde{t})$ in equation (41), and thus the slow noise could significantly affect the output (Figure S3F). Nevertheless, the leading order in (40) again matches the whole solution well (Figure S3C).

3. Noise is decoupled into a sum of fast and slow noise terms, i.e., $s = s_0 + s_1(t^+) + s_2(\tilde{t})$.

   In this case, we take b_0 as the solution of

$$\frac{db_0}{d\tilde{t}} = k_c s_2(\tilde{t})(1 - b_0)\frac{k_1 b_0 + k_3}{k_1 b_0 + k_2} - b_0 + k_4.$$

   Thus, only the slow term enters in the equation of b_0, and the fast noise is filtered out through the system (Figures S3G-S3J).

In summary, the zero-order solutions match the whole solution very well (Figures S3A-S3C). System (3) is not significantly affected by noise on a fast time scale (Figures S3D-S3E). The single-positive-loop system can function as a low-pass filter for different nature of noises (Figure S3D-S3J).

4.2 Connection to the linearization approach

The zero-order approximation obtained in (40) is a global solution, while the linear stability analysis in Sections 1-3 focuses on local solutions around the steady states. In this section, we use the deactivation process of the single-positive-loop system to elucidate the connections between these two approaches. Comparison to other systems could be done in a similar fashioin, but much more complicated computationally.

During deactivation, $s \equiv 0$ and the solution of (39) is

$$b_0(\tilde{t}) = (\beta_0 - k_4)e^{-\tilde{t}} + k_4.$$

Writing in the new coordinate $b_0^* = b_0 - k_4$, we have $b_0^*(\tilde{t}) = \beta_0^* e^{-\tilde{t}}$, where $\beta_0^* = \beta_0 - k_4$ is the initial condition. For the solution c_0 in (38), we linearize it around

$$\bar{c}_0 := \frac{k_1 k_4 + k_3}{k_1 k_4 + k_2},$$

obtaining

$$\begin{aligned} c_0^*(t^+, \tilde{t}) = &\left(\gamma_0^* - \beta_0^* \frac{(k_2 - k_3)k_1}{(k_1 k_4 + k_2)(k_1(\beta_0^* e^{-\tau_b t} + k_4) + k_2)} e^{-\tilde{t}}\right) e^{-(k_1(\beta_0^* e^{-\tilde{t}} + k_4) + k_2)t^+} \\ &+ \beta_0^* \frac{(k_2 - k_3)k_1}{(k_1 k_4 + k_2)(k_1(\beta_0^* e^{-\tau_b t} + k_4) + k_2)} e^{-\tilde{t}}, \end{aligned} \tag{42}$$

where $\gamma_0^* = \gamma_0 - \bar{c}_0$ is the initial condition of c_0^*. The dynamics of c_0^* is determined by two exponential functions. One is on the fast time scale t^+, and the other one is on the slow time scale $\tilde{t}$. Notice that (42) is in the same form of the equation (12), obtained using linearization and eigenvalue analysis ($\tilde{t} = \tau_b t$, $t^+ = t$).

5 Fluctuation-Dissipation Theorem Approach

Let us first review the Fluctuation-Dissipation Theorem used in [4, 5, 9, 10]. Consider a general input-output system

$$\frac{dx_i}{dt} = J_i^+(x) - J_i^-(x), \quad i = 1, \ldots, n. \tag{43}$$

Here, J_i^+ and J_i^- are the fluxes of the production and degradation of x_i, respectively. We assume that x_0 is the only source of noise, and $x_0(t) = \langle x_0 \rangle + \sigma_0(t)$, where σ_0 has mean zero and autocorrelation time τ_0. The two quantities that we are interested are the sensitivity of the steady state and the noise amplification

rate. We define the sensitivity of the steady state for each $i = 1, \ldots, n$ by *susceptibility* [5, 9, 10, 12]

$$s_i = \frac{\langle x_0 \rangle}{\langle x_i \rangle} \frac{d\langle x_i \rangle}{d\langle x_0 \rangle},$$

and the noise amplification rate by [5]

$$r_i = \frac{\eta_i}{\eta_0} = \frac{\mathrm{std}(x_i)/\langle x_i \rangle}{\mathrm{std}(x_0)/\langle x_0 \rangle}.$$

Both quantities are closely related to the *reaction flux elasticities* [5, 9, 10], H_{ij}, which measures how the ratio of production to degradation changes with respect to the concentrations,

$$H_{ij} = -\frac{\langle x_j \rangle}{\langle J_i \rangle} \left(\frac{\partial \langle J_i^+ \rangle}{\partial \langle x_j \rangle} - \frac{\partial \langle J_i^- \rangle}{\partial \langle x_j \rangle} \right) = \frac{\partial \ln \langle J_i^- \rangle}{\partial \ln \langle x_j \rangle} - \frac{\partial \ln \langle J_i^+ \rangle}{\partial \ln \langle x_j \rangle} = -\frac{\partial \ln \left(\langle J_i^+ \rangle / \langle J_i^- \rangle \right)}{\partial \ln \langle x_j \rangle}. \tag{44}$$

The susceptibility s_i can be solved from the equation [5, 9, 10]

$$H_{i0} + H_{i1} s_1 + \cdots + H_{in} s_n = 0, \quad i = 1, \ldots n. \tag{45}$$

The η_i's are solutions to the following equation [5, 9, 10]:

$$M\eta + \eta M^t + D = 0, \tag{46}$$

where

$$M_{ij} = -\frac{\langle J_i \rangle}{\langle x_i \rangle} \left(\frac{\partial \ln \langle J_i^- \rangle}{\partial \ln \langle x_j \rangle} - \frac{\partial \ln \langle J_i^+ \rangle}{\partial \ln \langle x_j \rangle} \right) = -\frac{\langle J_i \rangle}{\langle x_i \rangle} H_{ij}, \quad \eta_{ii} = \eta_i^2,$$

$$D_{00} = \frac{2\eta_0^2}{\tau_0}, \quad D_{ij} = 0 \text{ for } (i,j) \neq (0,0), i,j = 0, \ldots, n.$$

Applying to our single-positive-loop system (3), we have

$$n = 2, \quad x_0 = s, \quad x_1 = c, \quad x_2 = b,$$

$$J_1^+ = k_1 b(1-c) + k_3, \quad J_1^- = k_2 c, \quad J_2^+ = (k_c sc(1-b) + k_4)\tau_b, \quad J_2^- = b\tau_b.$$

By the definition of H_{ij} in (44), we obtain

$$H = (H_{ij}) = \begin{pmatrix} -1 & B & -1 \\ 0 & -1 & A/k_2 \end{pmatrix}, \quad i = 1, 2, \quad j = 0, 1, 2,$$

where $A = \langle \bar{b} \rangle k_1 + k_2, B = 1 + k_c \langle \bar{c} \rangle \langle s \rangle$. Thus,

$$M = (M_{ij}) = \begin{pmatrix} -\frac{1}{\tau_0} & 0 & 0 \\ \tau_b & -\tau_b B & \tau_b \\ 0 & k_2 & -A \end{pmatrix}, \quad \eta = (\eta_{ij}) = \begin{pmatrix} \eta_0^2 & \eta_{01} & \eta_{02} \\ \eta_{10} & \eta_1^2 & \eta_{12} \\ \eta_{20} & \eta_{21} & \eta_{22} \end{pmatrix},$$

$$D = (D_{ij}) = \begin{pmatrix} -\frac{2\eta_0^2}{\tau_0} & 0 & 0 \\ 0 & 0 & 0 \\ 0 & 0 & 0 \end{pmatrix}, \quad i = 0, 1, 2, \quad j = 0, 1, 2.$$

Notice that a key approximation in the FDT approach is that the average rates can be approximated by the rates at the average concentration [6, 9, 10]. Equations (45)-(46) can be solved explicitly, and we have

$$s_2 = \frac{k_2}{AB - k_2}, \quad r_2^2 = \frac{\eta_2^2}{\eta_0^2} = \frac{k_2^2 \tau_0 \tau_b (1 + A\tau_0 + B\tau_0\tau_b)}{(AB - k_2)(A + B\tau_b)(1 + A\tau_0 + B\tau_0\tau_b + \tau_0^2\tau_b(AB - k_2))}.$$

When $\tau_0\tau_b \ll 1$, we have $A\tau_0 \ll \tau_0^2\tau_b AB$, and thus,

$$r_2^2 \approx \frac{k_2^2 \tau_0 \tau_b}{(AB - k_2)(A + B\tau_b)}.$$

If further, $\tau_b \ll 1$,

$$r_2^2 \approx \frac{k_2^2 \tau_0 \tau_b}{(AB - k_2)A} \approx \frac{\tau_0 \tau_b}{\langle s \rangle (K_a k_c - 1)(K_a + 1)\frac{k_c}{k_c + 1}}. \tag{47}$$

Therefore, the amplification rate is a decreasing function in K_a and k_c (as $K_a k_c > 1$). Moreover, the noise amplification rate not only depends on τ_b, but also depends on K_a and k_c, in a way that larger K_a or k_c leads to better noise attenuation. The proposed quantity $(t_{1\to 0} - t_{0\to 1})$ incorporates both time scale of the b-equation (τ_b) and the key kinetic constants (k_c and K_a), and thus, serves as a better characterization than merely the time scale of the b-equation, τ_b. In our application, the autocorrelation time τ_0 is the inverse of the noise frequency, ω. Therefore, (47) becomes

$$r_2^2 \approx \frac{\tau_b}{\omega \langle s \rangle (K_a k_c - 1)(K_a + 1)\frac{k_c}{k_c+1}},$$

and the inverse relation between r_2 and ω arises naturally.

6 Models with Hill Functions

Hill functions are often used to model saturation effect and cooperativity in enzymatic reactions. In this section, we study the positive feedback module in Figure 1A based on Hill functions. First, we introduce Hill functions to the activation processes, as in the previous work of [2]:

$$\begin{aligned} \frac{dc}{dt} &= k_1(a+b)(1-c) - k_2 c + k_3, \\ \frac{da}{dt} &= (k_c s \frac{c^n}{c^n + K^n}(1-a) - a + k_4)\tau_a, \\ \frac{db}{dt} &= (k_c s \frac{c^n}{c^n + K^n}(1-b) - b + k_4)\tau_b. \end{aligned} \tag{48}$$

When the Hill exponent is one, system (48) corresponds to Michaelis-Menten kinetics, whereas system (16) is derived following mass action kinetics. In [2], $n = 3$ is used. Plotting the steady state response of the output as a function of constant input s for system (16) and system (48) with $n = 1$ and $n = 3$ yields three close curves (Figure S5A). Note that when n is large, for example, $n = 10$, bistability could arise in system (48), which is expected [1, 3]. Here, since we are solely interested in systems with a unique response to a constant signal, we focus on small n's, such as $n = 3$.

The time evolution of the outputs of the above three systems in response to a noise-free signal (Figure S5B) and the noisy signal (Figures S5C-S5D) shows extensive overlapping, suggesting similar relationship of the noise attenuation and the deactivation and activation time scales between system (48) and system (16). This is confirmed by direct simulation (Figure S5E-S5G). However, the analytical study for the model using Hill equations becomes more difficult, in particular, for Hill exponents larger than two.

Next, we considered a variation of system (48),

$$
\begin{aligned}
\frac{dc}{dt} &= k_1(a+b)(1-c) - k_2 c + k_3, \\
\frac{da}{dt} &= (k_c s \frac{c^n}{c^n + K^n}(1-a) - \frac{a^m}{a^m + L^m} + k_4)\tau_a, \\
\frac{db}{dt} &= (k_c s \frac{c^n}{c^n + K^n}(1-b) - \frac{b^m}{b^m + L^m} + k_4)\tau_b,
\end{aligned}
\tag{49}
$$

with nonlinear deactivation terms in A and B loops. The simulations of (49) with $L = 1$ and $m = 1$ show similar outputs with system (48) (Figures S6A-S6B). Furthermore, the noise amplification rate displays the same trend as in all other models (Figures S6C-S6E).

7 A Polymyxin B Resistance Model in Enteric Bacteria

We use the same equations as in [8] to describe the feedforward connector loop (FCL) model:

$$
\begin{aligned}
\frac{dx_1}{dt} &= k_{pbgP}\left(1 - \frac{1}{(1+K_2[PhoP\text{-}P]^2)(1+K_3(x_4^2+x_5^2))}\right) - k_{-pbgP}x_1 \\
\frac{dx_2}{dt} &= k_{PmrD}\frac{K_1[PhoP\text{-}P]^2}{1+K_1[PhoP\text{-}P]^2} - k_{-PmrD}x_2 - k_c x_2 x_4 + k_{-c}x_5 \\
\frac{dx_3}{dt} &= k_{PmrA} - k_{PmrA}x_3 + k_{-p}x_4 + k_p x_3 \\
\frac{dx_4}{dt} &= k_p x_3 - k_{-p}x_4 + k_{-c}x_5 - k_c x_2 x_4 \\
\frac{dx_5}{dt} &= k_c x_2 x_4 - k_{-c}x_5.
\end{aligned}
\tag{50}
$$

The parameter values used in simulations are listed in Table S1.

References

[1] D. Angeli, J. E. Ferrell, and E. D. Sontag. Detection of multistability, bifurcations, and hysteresis in a large class of biological positive-feedback systems. *Proc. Natl. Acad. Sci. USA*, 101(7):1822–1827, 2004.

[2] O. Brandman, J. E. Ferrell, R. Li, and T. Meyer. Interlinked fast and slow positive feedback loops drive reliable cell decisions. *Science*, 310(5747):496–498, 2005.

[3] J. L. Cherry and F. R. Adler. How to make a biological switch. *J Theor Biol*, 203:117–133, 2000.

[4] C. W. Gardiner. *Handbook of Stochastic Methods.* Springer, third edition, 2004.

[5] G. Hornung and N. Barkai. Noise propagation and signaling sensitivity in biological networks: a role for positive feedback. *PLoS Comput Biol.*, 4(1):e8, 2008.

[6] J. E. Keizer. *Statistical Thermodynamics of Nonequilibrium Processes.* Springer-Verlag, New York, New York, 1987.

[7] J. Kevorkian and J. D. Cole. *Perturbation Methods in Applied Mathematics*, volume 34 of *Applied Mathematical Sciences.* Springer-Verlag, Berlin and New York, 1981.

[8] A.Y. Mitrophanov, M.W. Jewett, T.J. Hadley, and E.A. Groisman. Evolution and dynamics of regulatory architectures controlling polymyxin B resistance in enteric bacteria. *PLoS Genetics*, 4(10), 2008.

[9] J. Paulsson. Summing up the noise in gene networks. *Nature*, 427:415–418, 2004.

[10] J. Paulsson. Models of stochastic gene expression. *Phys. Life Rev.*, 2:157–75, 2005.

[11] Rüdiger Seydel. *Practical Bifurcation and Stability Analysis: From Equilibrium to Chaos*. Springer-Verlag, second edition, 1994.

[12] P. S. Swain, M. B. Elowitz, and E. D. Siggia. Intrinsic and extrinsic contributions to stochasticity in gene expression. *Proc Natl Acad Sci U S A*, 99:12795–12800, 2002.

[13] X. P. Zhang, Z. Cheng, F. Liu, and W. Wang. Linking fast and slow positive feedback loops creates an optimal bistable switch in cell signaling. *Physical Review E*, 76:031924, 2007.
