# Supplementary material for: A Critical Quantity for Noise Attenuation in Feedback Systems
Source: PLoS Comput Biol. 2010 Apr 29;6(4):e1000764. doi: 10.1371/journal.pcbi.1000764 (PMC2861702; doi:10.1371/journal.pcbi.1000764)
Supplement: Figure S1 — Noise amplification rate in single-positive-loop systems with respect to t1→0 and t0→1, respectively. (0.08 MB PDF) [file pcbi.1000764.s002.pdf]

## Figure S1

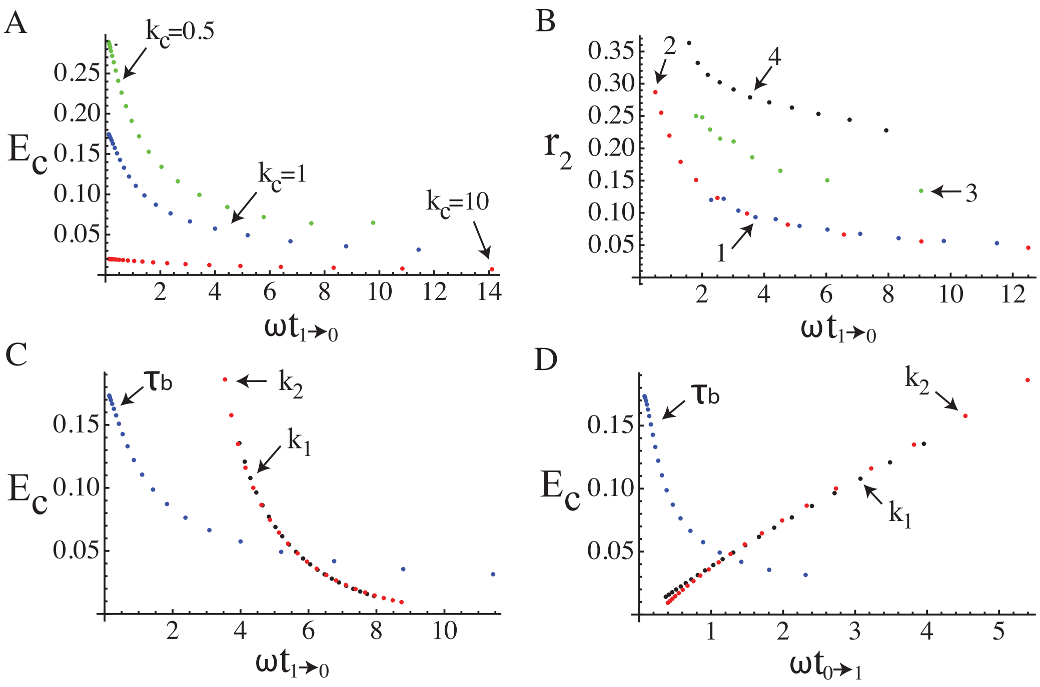


**Figure S1: Noise amplification rate in single-positive-loop systems with respect to $t_{1\to0}$ and $t_{0\to1}$, respectively.** (A) $E_c$ versus $\omega t_{1\to0}$ for $k_c = 0.5, 1, 10$. Each dotted curve consists of 21 simulations with $(\tau_b)_n = 0.005e^{\Delta\tau_b n}$ and $\Delta\tau_b = -\ln(0.005)/20$. (B) $r_2$ versus $\omega t_{1\to0}$. Each point represents an average of $r_2$ based on 100 simulations with different noisy signals but fixed $\omega$. Set 1 (blue): $\tau_b = 0.01$, $k_1 = 3$, $k_2 = 0.3$. Set 2 (red): $\tau_b = 0.1$, $k_1 = 3$, $k_2 = 0.3$. Set 3 (green): $\tau_b = 0.01$, $k_1 = 3$, $k_2 = 0.6$. Set 4 (black): $\tau_b = 0.01$, $k_1 = 1$, $k_2 = 0.3$. In set 2, $\omega$ takes $(1/\omega)_m = 2e^{\Delta dm}$, $\Delta d = \ln(50/2)/10$. For the rest, $(1/\omega)_m = 20e^{\Delta dm}$, $\Delta d = \ln(100/20)/10$. (C-D) $E_c$ versus $\omega t_{1\to0}$ (C) or $\omega t_{0\to1}$ (D). $\tau_b$ curve (blue): $(\tau_b)_n = 0.005e^{\Delta\tau_b n}$, $\Delta\tau_b = -\ln(0.005)/20$; $k_2$ curve (red): $(k_2)_n = 0.06e^{\Delta k_2 n}$, $\Delta k_2 = \ln(1.2/0.06)/20$; $k_1$ curve (black): $(k_1)_n = e^{\Delta k_1 n}$, $\Delta k_1 = \ln(10)/20$. All simulations use the same parameters and inputs as their counterparts in Figure 3, unless otherwise specified.
