# Supplementary material for: A Critical Quantity for Noise Attenuation in Feedback Systems
Source: PLoS Comput Biol. 2010 Apr 29;6(4):e1000764. doi: 10.1371/journal.pcbi.1000764 (PMC2861702; doi:10.1371/journal.pcbi.1000764)
Supplement: Figure S2 — Noise amplification rate in positive-positive-loop systems with respect to t1→0 and t0→1, respectively. (0.07 MB PDF) [file pcbi.1000764.s003.pdf]

Figure S2

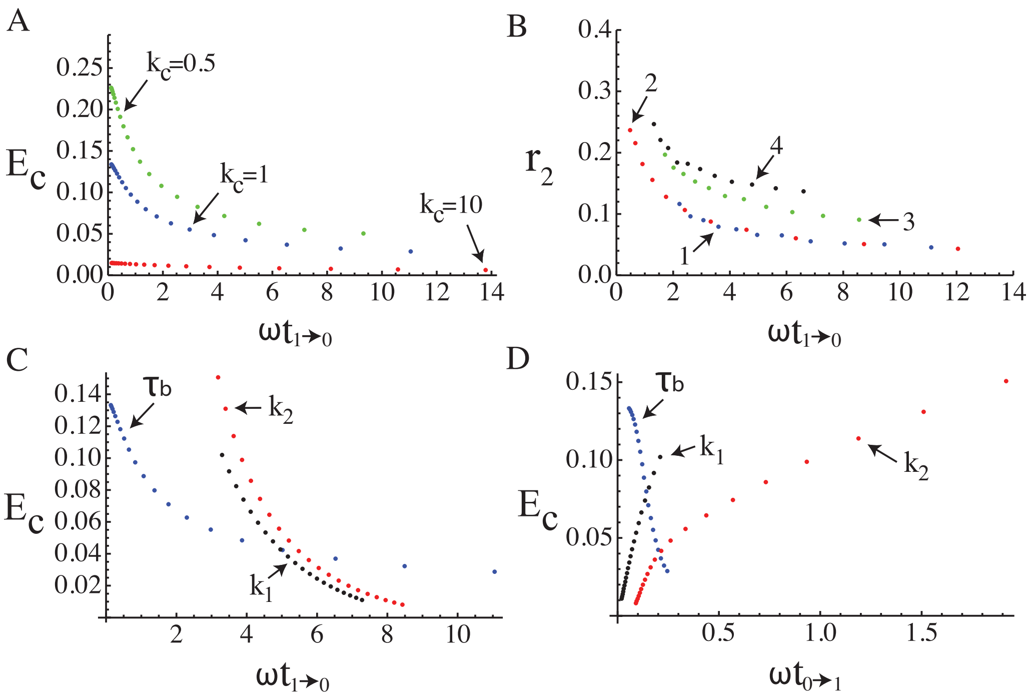

Figure S2: Noise amplification rate in positive-positive-loop systems with respect to $t_{1\to0}$ and $t_{0\to1}$, respectively. (A) E_c versus $\omega t_{1\to0}$ for $k_c = 0.5, 1, 10$. (B) r_2 versus $\omega t_{1\to0}$. (C-D) E_c versus $\omega t_{1\to0}$ (C) or $\omega t_{0\to1}$ (D). All simulations use the same parameters and inputs as their counterparts in Figure S1 with the additional parameter $\tau_a = 1$, unless otherwise specified.
