# Supplementary material for: A Critical Quantity for Noise Attenuation in Feedback Systems
Source: PLoS Comput Biol. 2010 Apr 29;6(4):e1000764. doi: 10.1371/journal.pcbi.1000764 (PMC2861702; doi:10.1371/journal.pcbi.1000764)
Supplement: Figure S3 — Two-time-scale decomposition of the single-positive-loop system (1) in the main text. (0.23 MB PDF) [file pcbi.1000764.s004.pdf]

Figure S3

Figure S3: Two-time-scale decomposition of the single-positive-loop system (1) in the main text. (A)-(C) The zero-order approximation (dashed line) and the whole solution (solid line) of c (red) and b (black) in response to the signal $s(t) = 1 + \sin(2\pi\omega t)$. (A) High frequency, $\omega = 1$. (B) Medium frequency, $\omega = 0.1$. (C) Low frequency, $\omega = 0.01$. (D)-(F) The noise-free approximation (blue) versus zero-order approximation (red) of c in response to the signal $s(t) = 1 + \sin(2\pi\omega t)$. (D) High frequency, $\omega = 1$. (E) Medium frequency, $\omega = 0.1$. (F) Low frequency, $\omega = 0.01$. (G) The slow quasi-periodic noise profile, $s(t) = 1 + (\sin(2\pi\omega t) + \sin(2\sqrt{2}\pi\omega t))/2, \omega = 0.01$. (H) The noise profile of $s(t) = 1 + \sum_1^{2000} \frac{\xi_k}{k} \sin(2\pi k\omega t)$, $\xi_k \sim N(0,1)$, $\omega = 0.01$. (I) The corresponding output of (G). (J) The corresponding output of (H). All simulations use the same parameters as in Figure 3, unless otherwise specified. The initial condition is $(c, b) = (0.1, 0)$.

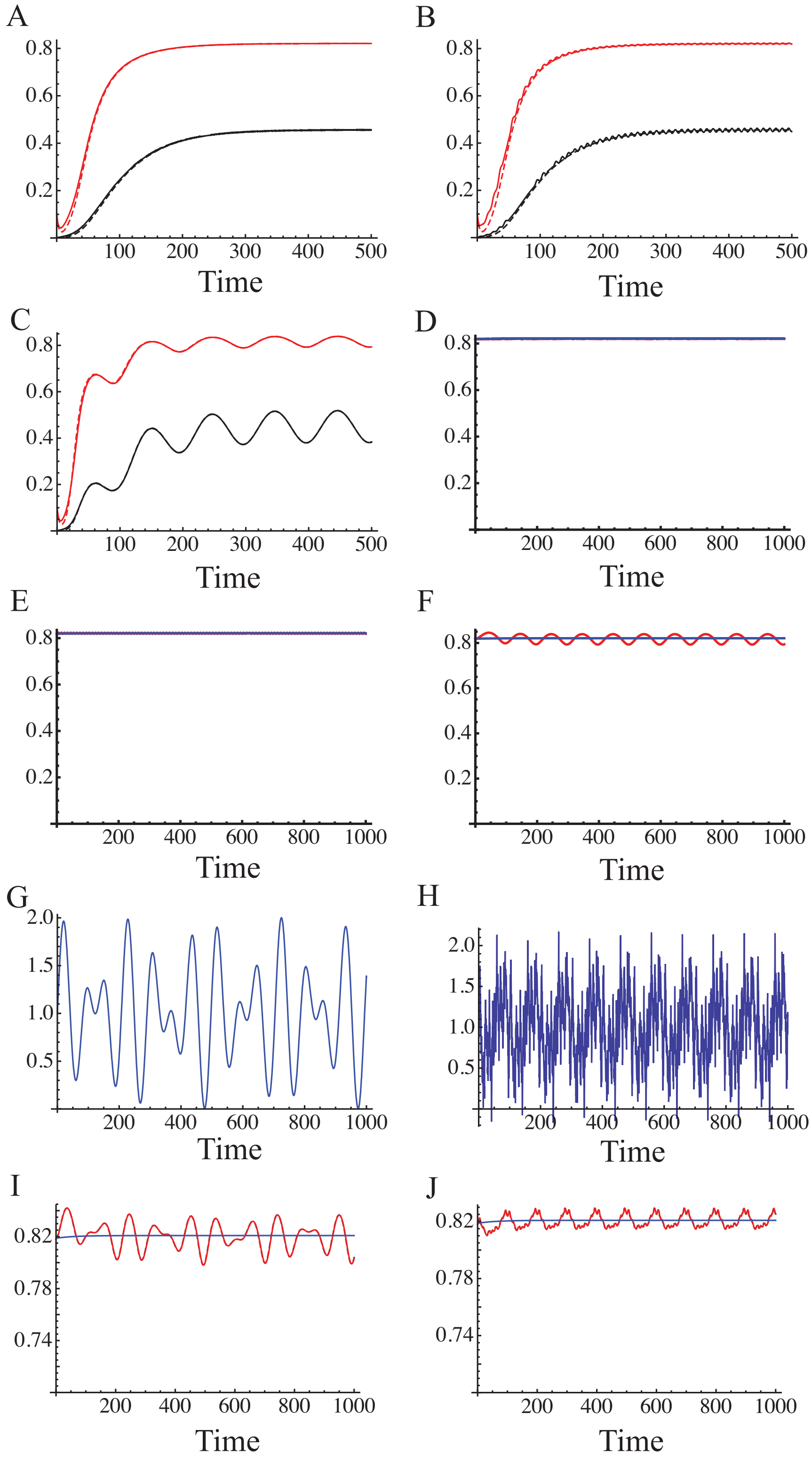
A
B
C
D
E
F
G
H
I
J
Time
