# Supplementary material for: A Critical Quantity for Noise Attenuation in Feedback Systems
Source: PLoS Comput Biol. 2010 Apr 29;6(4):e1000764. doi: 10.1371/journal.pcbi.1000764 (PMC2861702; doi:10.1371/journal.pcbi.1000764)
Supplement: Figure S4 — The ratio of noise amplification rates in positive-negative-loop systems to single-positive-loop systems. (0.03 MB PDF) [file pcbi.1000764.s005.pdf]

Figure S4

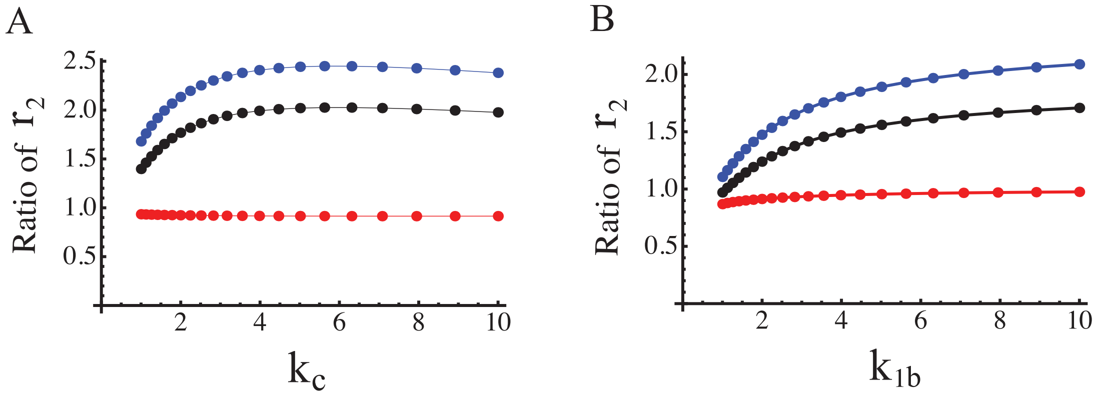

Figure S4: The ratio of noise amplification rates in positive-negative-loop systems to single-positive-loop systems. (A-B) Each dot represents the ratio of noise amplification rates in a positive-negative-loop system to the corresponding single-positive-loop system at a particular k_c (A) or k_{1b} (B) value. Red: $\tau_a = 0.01$; black: $\tau_a = 0.1$, blue: $\tau_a = 1$. Other parameters are the same as in Figure 5.
