# Supplementary material for: A Critical Quantity for Noise Attenuation in Feedback Systems
Source: PLoS Comput Biol. 2010 Apr 29;6(4):e1000764. doi: 10.1371/journal.pcbi.1000764 (PMC2861702; doi:10.1371/journal.pcbi.1000764)
Supplement: Figure S5 — Simulations of the Hill function model (46) in Text S1. (0.10 MB PDF) [file pcbi.1000764.s006.pdf]

## Figure S5

**Figure S5: Simulations of the Hill function model (46) in Text S1.** (A) Steady state responses to constant $s$. (B) Time evolution of the outputs of system (46) in Text S1 with $n = 3$ (blue dahsed) and system (14) in Text S1 (red solid) in response to the input (black solid). (C)-(D) Outputs of system (46) in Text S1 with $n = 3$ (blue dahsed) and system (14) in Text S1 (red solid) in response to the noisy signal in Figure 3A. In (C), $\tau_a = 1, \tau_b = 0.01$; in (D), $\tau_a = \tau_b = 1$. In (A)-(D), parameters are chosen the same as in the reference [30] of the main text. Specifically, where $\tau_b = 0.008$, $\tau_a = 0.5$, $k_1 = 2$, $k_2 = 0.3$, $k_3 = 0.001$, $k_4 = 0.01$, $n = 3$, $K = 0.35$, $k_c = 1$. (E) $r_2$ versus $t_{1\to 0}\omega$. (F) $r_2$ versus $t_{0\to 1}\omega$. (G) $r_2$ versus $(t_{1\to 0} - t_{0\to 1})\omega$. In (E)-(G), parameters are varied around those in (A)-(D). $\tau_b$ curve (blue): $(\tau_b)_n = 0.004e^{\Delta\tau_b n}, \Delta\tau_b = \ln(20)/5$; $k_2$ curve (red): $(k_2)_n = 0.06e^{\Delta k_2 n}, \Delta k_2 = \ln(10)/5$; $k_1$ curve (black): $(k_1)_n = e^{\Delta k_1 n}, \Delta k_1 = \ln(10)/5$; $k_c$ curve (green): $(k_c)_n = 0.5e^{\Delta\tau_a n}, \Delta\tau_a = \ln(20)/5$. In all simulations, $T_1, T_2, T_{\max}, t_1, t_2$ take the same values as in Figure 3.

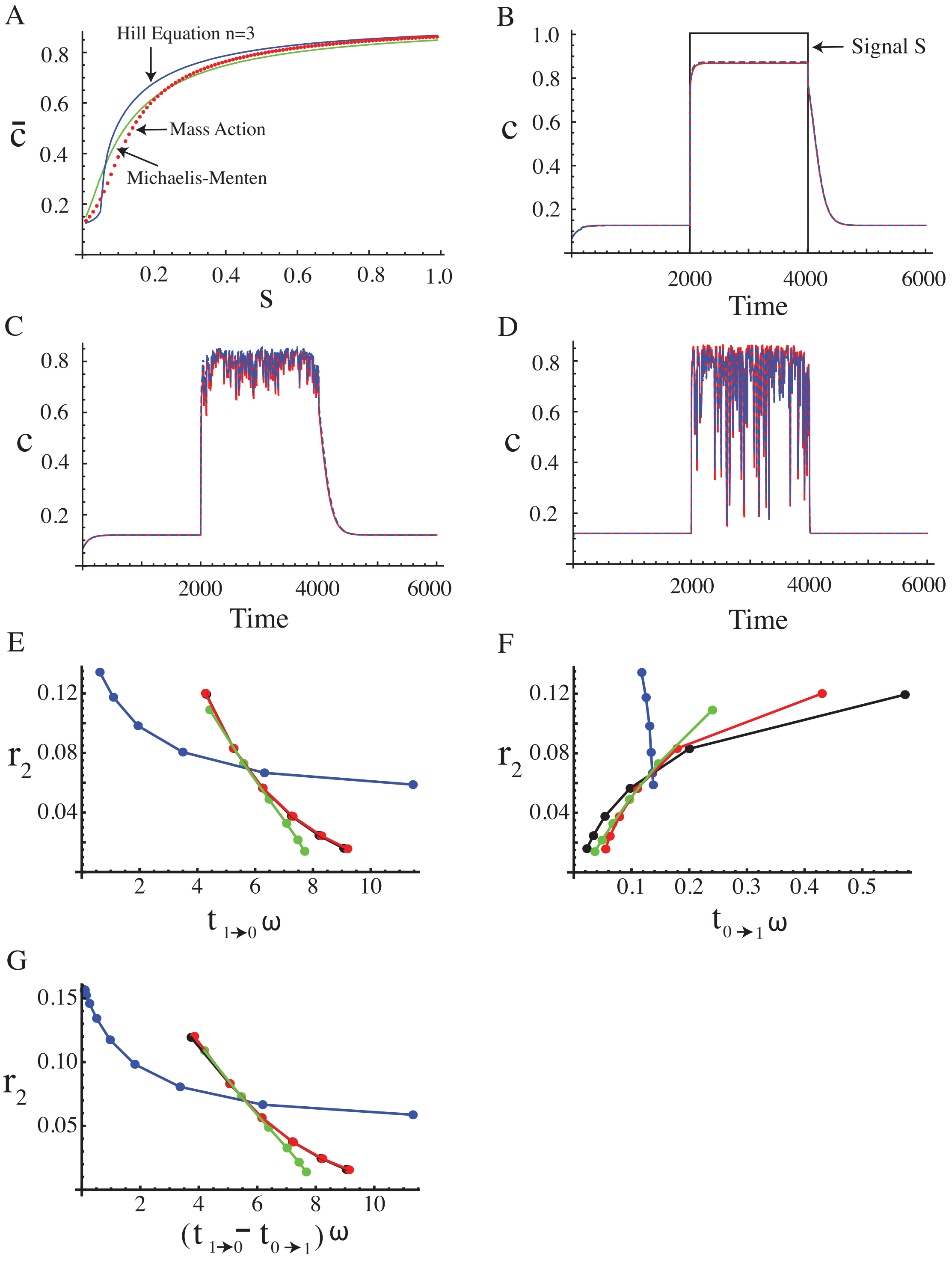

A
Hill Equation n=3
Mass Action
Michaelis-Menten
c̄
S
B
Signal S
c
Time
C
c
Time
D
c
Time
E
$r_2$
$t_{1\to0}\,\omega$
F
$r_2$
$t_{0\to1}\,\omega$
G
$r_2$
$(t_{1\to0} - t_{0\to1})\,\omega$
