# Supplementary material for: A Critical Quantity for Noise Attenuation in Feedback Systems
Source: PLoS Comput Biol. 2010 Apr 29;6(4):e1000764. doi: 10.1371/journal.pcbi.1000764 (PMC2861702; doi:10.1371/journal.pcbi.1000764)
Supplement: Figure S6 — Simulations of the Hill function model (47) in Text S1. (0.07 MB PDF) [file pcbi.1000764.s007.pdf]

Figure S6

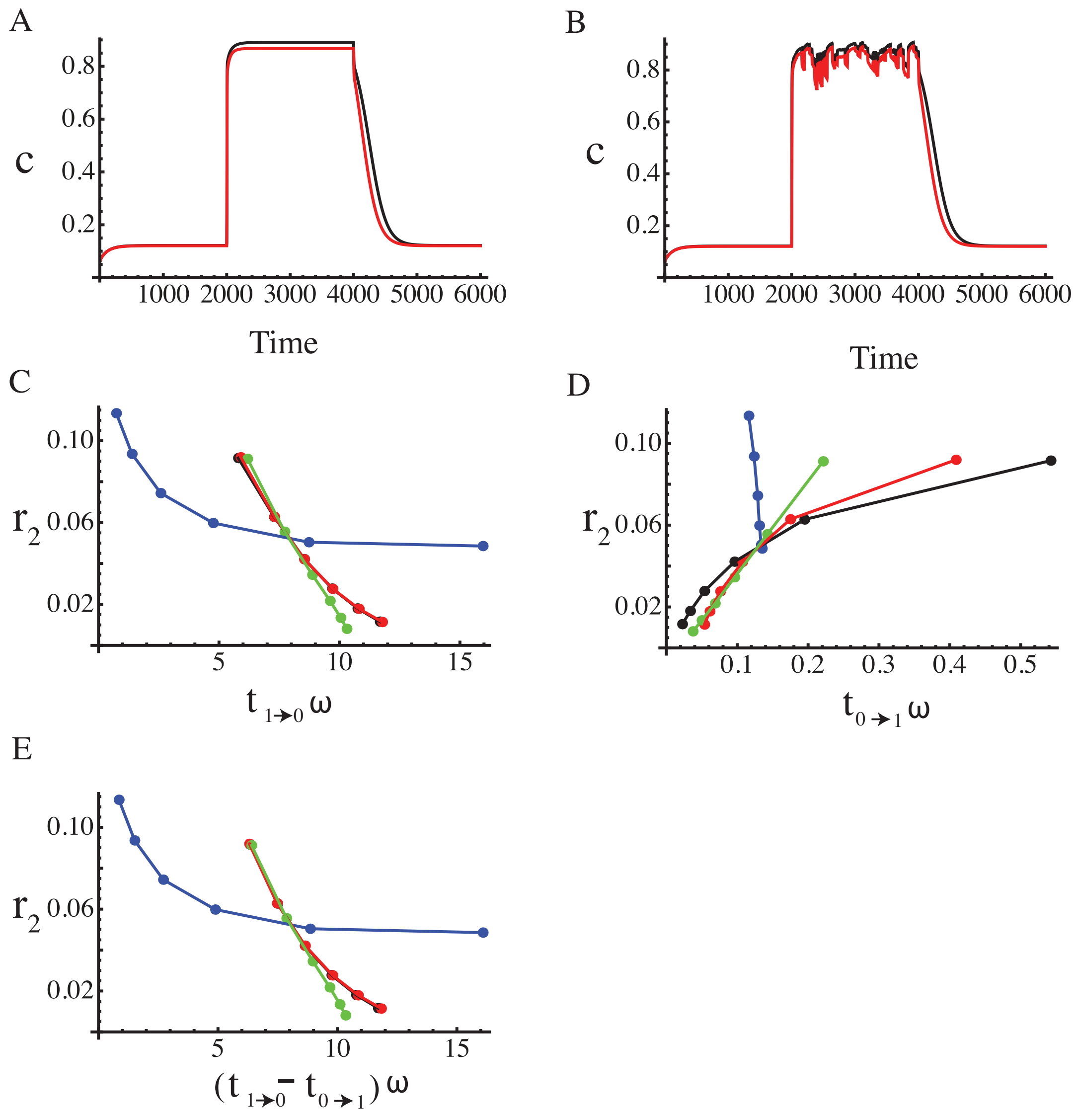

Figure S6: Simulations of the Hill function model (47) in Text S1. (A) The outputs of system (47) (black) and system (46) (red) in response to the pulse input in Figure S5B (black). (B) The outputs of system (47) (black) and system (46) (red) given the noisy input in Figure 3A. (C) The noise amplification rate versus $t_{1\to0}\omega$. (D) The noise amplification rate versus $t_{0\to1}\omega$. (E) The noise amplification rate versus $(t_{1\to0} - t_{0\to1})\omega$. In all simulations, $L = m = 1$, and all other parameters take the same values as in Figure S5.
