# Supplementary material for: A Critical Quantity for Noise Attenuation in Feedback Systems
Source: PLoS Comput Biol. 2010 Apr 29;6(4):e1000764. doi: 10.1371/journal.pcbi.1000764 (PMC2861702; doi:10.1371/journal.pcbi.1000764)
Supplement: Figure S7 — The full plot of Figure 6G. (0.03 MB PDF) [file pcbi.1000764.s008.pdf]

Figure S7

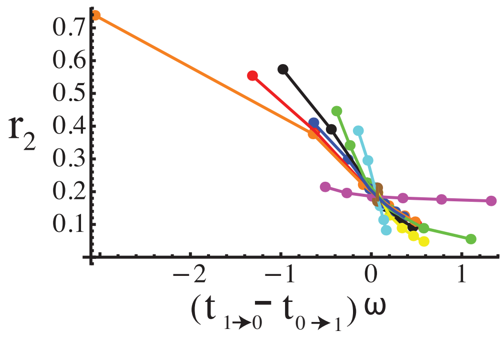

Figure S7: The full plot of Figure 6G. Ten parameters are varied systematically in ±3-fold ranges based on their original values given in (D). Each variation corresponds to one curve on the plot. The ten parameters are k_{42a} (red), k_{42d} (black), k_{24d} (pink), k_{24cm0} (magenta), k_{24cm1} (yellow), k_{24mc} (orange), k_{RL} (cyan), k_{RLm} (green), k_{B1cm} (blue), k_{Cla4a} (brown).
