# Supplementary material for: A Critical Quantity for Noise Attenuation in Feedback Systems
Source: PLoS Comput Biol. 2010 Apr 29;6(4):e1000764. doi: 10.1371/journal.pcbi.1000764 (PMC2861702; doi:10.1371/journal.pcbi.1000764)
Supplement: Figure S8 — Simulations of the four connector-mediated models. The activation (A) and deactivation (B) dynamics of the regulator-protecting (RP) model (blue), the regulator-activating (RA) model (green), the phosphatase-inhibiting (PI) model (black), and the kinase-stimulating (KS) model (red). (C–F) The output of the RP model (C), the RA model (D), the PI model (E), and the KS model (F). In (C–F), we use the same input (G). (0.09 MB PDF) [file pcbi.1000764.s009.pdf]

Figure S8

Figure S8: Simulations of the four connector-mediated models. The activation (A) and deactivation (B) dynamics of the regulator-protecting (RP) model (blue), the regulator-activating (RA) model (green), the phosphatase-inhibiting (PI) model (black), and the kinase-stimulating (KS) model (red). (C-F) The output of the RP model (C), the RA model (D), the PI model (E), and the KS model (F). In (C-F), we use the same input (G).

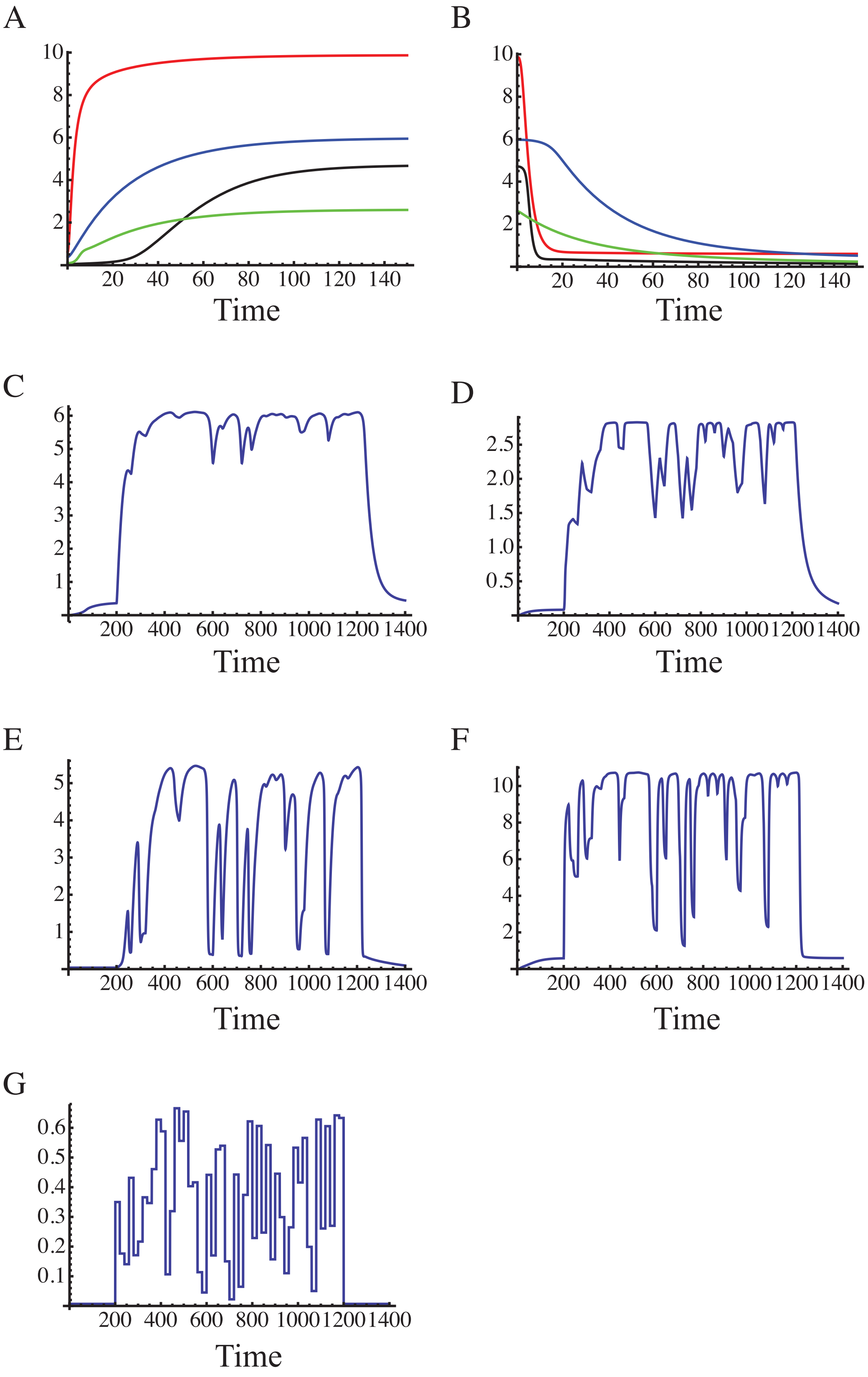
A
B
C
D
E
F
G
Time
