# Supplementary material for: A Critical Quantity for Noise Attenuation in Feedback Systems
Source: PLoS Comput Biol. 2010 Apr 29;6(4):e1000764. doi: 10.1371/journal.pcbi.1000764 (PMC2861702; doi:10.1371/journal.pcbi.1000764)
Supplement: Table S1 — Parameters used in the simulation of the polymyxin B resistance model. The values of kp and k−p correspond to kpmax = 1, k−pmax = 2, and f = 0.05 in [38], a case of mild activation from the second input. (0.05 MB PDF) [file pcbi.1000764.s010.pdf]

Table S1

Parameters	Units	Values
k_{pbgP}	μM/min	0.05
k_{-pbgP}	min^{-1}	0.1
k_{PmrD}	μM/min	10
k_{-PmrD}	min^{-1}	0.1
k_c	1/(min$\cdot\mu$M)	5
k_{-c}	min^{-1}	0.5
k_{PmrA}	μM/min	0.05
k_{-PmrA}	min^{-1}	0.1
k_p	min^{-1}	0.05
k_{-p}	min^{-1}	1.9
K_1	$(\mu M)^{-2}$	1
K_2	$(\mu M)^{-2}$	1
K_3	$(\mu M)^{-2}$	1

Table S1: Parameters used in the simulation of the polymyxin B resistance model. The values of k_p and k_{-p} correspond to $k_p^{max} = 1$, $k_{-p}^{max} = 2$, and $f = 0.05$ in [1], a case of mild activation from the second input.

References

[1] A.Y. Mitrophanov, M.W. Jewett, T.J. Hadley, and E.A. Groisman. Evolution and dynamics of regulatory architectures controlling polymyxin B resistance in enteric bacteria. *PLoS Genetics*, 4(10), 2008.
